# Integrated Multi-Omics Analysis Reveals Associations Between Flavor Variation, Bacterial Communities, Metabolites, and Lipids in Commercial Dry-Cured Grass Carp

**DOI:** 10.3390/foods15193547

**Published:** 2026-10-04

**Authors:** Tianxiao Zhang, Ji Wang, Xuhui Huang, Lili Zhang, Xuechen Pei

**Affiliations:** 1 Jinzhou Medical University, Jinzhou 121001, China; mm217a@126.com; 2 First Affiliated Hospital, Jinzhou Medical University, Jinzhou 121001, China; 3 School of Food Science and Technology, SKL of Marine Food Processing & Safety Control, Collaborative Innovation Center of Seafood Deep Processing, National Engineering Research Center of Seafood, Dalian Polytechnic University, Dalian 116034, China; 17835422765@163.com (J.W.); xuhuisos@dlpu.edu.cn (X.H.); 4 College of Food and Health, Jinzhou Medical University, Jinzhou 121001, China; 5 Liaoning Provincial Professional Technology Innovation Center of Meat Processing and Quality-Safety Control, Jinzhou 121001, China

**Keywords:** grass carp, volatiles, microorganisms, lipids, metabolites

## Abstract

Commercial dry-cured grass carp products exhibit considerable flavor variation, but the microbial and biochemical characteristics associated with this variation remain unclear. In this study, six commercial products were comparatively characterized using electronic nose and tongue analyses, GC-MS, full-length 16S rRNA gene sequencing, metabolomics, and lipidomics. Among the six sampled products, Group F exhibited distinct aroma and taste profiles and showed a greater diversity and higher measured levels of volatile compounds on a wet-weight basis, with 1-octen-3-ol, hexanal, nonanal, and (*E*)-4-decenal among its prominent aroma-active compounds. Bacterial community structures differed significantly among the sampled products, with distinct species-level profiles; notably, *Edwardsiella piscicida* predominated in Group D, whereas *Staphylococcus equorum* and *Staphylococcus xylosus* were abundant in Groups E and F. Metabolomic and lipidomic analyses further revealed pronounced product-specific differences, particularly in organic acid- and amino acid-related metabolites and in glycerolipid and glycerophospholipid profiles. Exploratory correlations suggested potential product-level associations among bacterial taxa, lipid and metabolite profiles, and aroma-active volatiles, although none remained statistically significant after FDR correction. Overall, the results reveal coordinated differences in flavor, bacterial communities, metabolites, and lipids among the six commercial dry-cured grass carp products examined in this study and provide a compositional basis for further investigation of flavor formation.

## 1. Introduction

Grass carp (*Ctenopharyngodon idella*) is one of the most important freshwater aquaculture species worldwide [1,2]. It is also valued for its nutritional quality, including high-quality protein and a favorable fatty acid composition [3]. In China, dry-curing is an important approach for processing grass carp, in which salting and dehydration contribute to preservation and the development of characteristic flavor [4]. However, commercially available dry-cured grass carp products exhibit considerable diversity in their commercial sources, raw materials, and processing characteristics, which may contribute to variations in flavor profiles. As flavor is a major determinant of consumer acceptance and product quality, investigating the factors associated with these variations is important for quality evaluation and process optimization.

Flavor is a complex attribute determined by both volatile and non-volatile compounds and is closely associated with biochemical and microbial transformations during processing [5]. Previous studies have characterized the volatile profiles of grass carp using instrumental approaches. Yu et al. [6] identified 23 volatile compounds in fresh grass carp fillets by gas chromatography-mass spectrometry (GC-MS), whereas Li et al. [4] identified 24 aroma-active compounds with odor activity values (OAVs) > 1 in low-salt dry-cured grass carp. Flavor formation is closely related to changes in flavor precursors. Lipid oxidation and degradation can generate aldehydes and other volatile compounds, with fatty acids serving as important aroma precursors [7], while amino acids, nucleotides, and organic acids contribute substantially to non-volatile taste characteristics [8]. In addition, microbial metabolism can promote protein and lipid degradation and consequently modify the formation of flavor compounds in processed fish [9]. Nevertheless, previous studies have mainly focused on individual classes of flavor compounds or specific processing conditions, and the factors underlying flavor differences among commercial dry-cured grass carp products remain poorly understood.

Because flavor development involves interconnected changes in microorganisms, metabolites, and lipids, analysis of a single group of compounds is insufficient to explain its formation. Combining microbial community profiling with metabolomics and lipidomics provides an opportunity to explore associations among bacterial communities, metabolites, lipids, and flavor variation. Combined with electronic sensing and GC-MS-based volatile analysis, such a multi-omics profiling strategy can therefore provide a broader view of the associations among microbial communities, metabolites, lipids, and flavor variation in dry-cured grass carp products.

In this study, six commercially available dry-cured grass carp products were investigated. Electronic nose and electronic tongue analyses, GC-MS, and OAV analysis were used to characterize their flavor profiles and identify key volatile compounds, while bacterial communities, non-volatile metabolites, and lipid profiles were further characterized using 16S rRNA gene sequencing and ultra-performance liquid chromatography-mass spectrometry (UPLC-MS). Correlation analyses were subsequently performed to explore potential associations among key volatile compounds, bacterial taxa, metabolites, and lipids, and to identify candidate factors potentially related to flavor variation among the commercial products. These findings provide insights into the compositional characteristics associated with flavor differences in dry-cured grass carp and may serve as a reference for flavor quality evaluation and further studies on process optimization.

## 2. Materials and Methods

### 2.1. Materials

Six commercially available dry-cured grass carp (*Ctenopharyngodon idella*) products were purchased from Taobao and designated as Groups A-F. According to the product-label information, the products were associated with Dongtai (Jiangsu, China), Jingzhou (Hubei, China), Ezhou (Hubei, China), Jiangmen (Guangdong, China), Liuan (Anhui, China), and Heyuan (Guangdong, China), respectively. After purchase, all samples were stored at −18 °C. These geographical locations were recorded only as descriptive information on product source and were not treated as an independent experimental factor. For each product, five unopened packages were independently purchased, with each package containing one individual fish. One dorsal-muscle sample was independently collected from each fish, and each individual fish was considered as one biological replicate. Three or four biological replicates were used for different analytical platforms. Due to differences in analytical requirements among platforms, the number of biological replicates varied slightly between analyses, including electronic nose (*n* = 3), electronic tongue (*n* = 4), GC-MS (*n* = 4), microbiome sequencing (*n* = 3), metabolomics (*n* = 3), and lipidomics (*n* = 3) analyses. No samples were pooled before microbiome, metabolomic, or lipidomic analyses. Because detailed production and lot information was unavailable, independence among samples at the production-batch level could not be confirmed. Accordingly, the study was interpreted primarily as a comparison among the sampled commercial products rather than as an assessment of generalizable production- or batch-level effects (Table S1).

According to the available product-label information, grass carp and salt were listed as the main raw materials. However, complete information on product formulations, possible seasonings or other added ingredients, salt addition levels, storage history, and detailed processing procedures, including drying, smoking, or fermentation conditions, was not available for these commercial products. Therefore, product identity, geographical source, and processing characteristics could not be independently separated in the present study, as these factors were inherently confounded among the commercial products. Accordingly, the observed differences among groups were interpreted as variations among the sampled commercial products rather than as direct effects attributable solely to geographical origin, formulation, or processing method.

### 2.2. Electronic Nose Analysis

An electronic nose (PEN 3; AIRSENSE Analytics GmbH, Schwerin, Germany) equipped with an array of 10 metal oxide semiconductor sensors (Table S2) was used [10]. The samples were pretreated as follows: 5 g of minced fish muscle (without cooking treatment) was transferred into a 20 mL sealed screw-cap vial, heated at 60 °C for 30 min, and subsequently equilibrated at room temperature for 20 min. The electronic nose parameters were set as follows: sampling interval, 1 s; pre-sampling time, 6 s; zero-point calibration time, 10 s; detection time, 60 s; cleaning time, 150 s; and injection flow rate, 200 mL/min.

### 2.3. Electronic Tongue Analysis

An electronic tongue (TS-5000Z, Insent Inc., Atsugi, Japan) equipped with five artificial lipid/polymer membrane sensors for detecting sourness, umami, bitterness, astringency, and saltiness was used to characterize the taste response patterns of dry-cured grass carp samples [11]. Raw dry-cured grass carp samples without cooking treatment were used for electronic tongue analysis. Briefly, 20 g of minced fish muscle was mixed with 100 mL of deionized water and homogenized at 8000 rpm for 50 s at 4 °C. The homogenate was then incubated at 40 °C for 50 min, followed by centrifugation at 2687× *g* for 5 min at 4 °C. The resulting supernatant was collected and filtered for subsequent electronic tongue analysis.

### 2.4. Analysis of Volatile Compounds

Volatile compounds were extracted using a headspace solid-phase microextraction (HS-SPME), which was equipped with a DVB/CAR/PDMS extraction fiber (divinylbenzene/carboxen/polydimethylsiloxane; Supelco, Bellefonte, PA, USA) [12]. Specifically, 4 g of minced fish muscle was placed in a 20 mL headspace vial, and 10 μL of cyclohexanone solution (50 μg/mL; cyclohexanone purity ≥ 99.5%; Aladdin Reagent Co., Shanghai, China) was added as an internal standard. After equilibration at 60 °C for 50 min, HS-SPME was performed for 40 min.

Volatile compounds were analyzed using a 7890B gas chromatograph coupled to a 5977A mass selective detector (GC-MS; Agilent Technologies Co., Ltd., Santa Clara, CA, USA) equipped with a non-polar HP-5MS capillary column (30 m × 0.25 mm × 0.25 μm). After HS-SPME extraction, the fiber was introduced into the GC injection port and thermally desorbed at 250 °C for 5 min. The GC-MS operating conditions were set according to Wang et al. [13].

Volatile compounds were tentatively identified by comparing their mass spectra with the NIST library and matching their calculated retention indices (RIs) with published reference values. The RIs were calculated using a homologous series of n-alkanes (C7-C30) as external standards. Since authentic chemical standards were not analyzed in the present study, none of the volatile compounds were assigned as MSI level 1 identifications. Compounds with both NIST spectral matching and RIs agreement were considered MSI level 2 identifications. Procedural blanks were analyzed using the same GC-MS procedure. Quantification was performed using the internal standard method according to Equation (1). In addition, the OAV of each volatile compound was calculated using Equation (2). Because the odor thresholds used for OAV calculation were generally determined in water, the effects of the salty and lipid-rich fish matrix on volatile partitioning and release were not fully accounted for. Thus, the OAVs should be regarded as approximate indicators of potential aroma contribution rather than absolute measures of sensory importance.

*C_x_* = *A_x_* × *C_i_* × *V*/(*A_i_* × *M*) (1)

where *C_x_* and *C_i_* represent the concentrations of volatile compound *x* and cyclohexanone, respectively; *A_x_* and *A_i_* represent the corresponding peak areas of volatile compound *x* and cyclohexanone, respectively; and *V* and *M* represent the volume of cyclohexanone and the mass of fish muscle, respectively.

*OAV_x_* = *C_x_*/*T_x_* (2)

where *OAV_x_* is the odor activity value of volatile compound *x*, *C_x_* represents the concentration of volatile compound *x*, and *T_x_* represents the odor threshold value of volatile compound *x* acquired from a reference [14].

### 2.5. Analysis of Bacterial Community

Bacterial diversity and community composition were analyzed according to the method described by Wang et al. [15], with minor modifications. Total genomic DNA was extracted from 0.5 g of dry-cured grass carp samples using the E.Z.N.A.™ Mag-Bind Soil DNA Kit (Omega Bio-tek, Norcross, GA, USA) according to the manufacturer’s instructions. A total of 18 biological samples were subjected to bacterial community analysis. The concentration of the extracted DNA was determined using a Qubit fluorometer (Thermo Fisher Scientific, Waltham, MA, USA), and DNA quality was evaluated prior to PCR amplification. The full-length bacterial 16S rRNA gene was amplified using the universal forward primer 5′-AGRGTTYGATYMTGGCTCAG-3′ and reverse primer 5′-RGYTACCTTGTTACGACTT-3′. PCR amplification was performed using 2× Hieff^®^ Robust PCR Master Mix (Yeasen Biotechnology Co., Shanghai, China). The PCR products were purified and used for library preparation. Library quality was assessed by 2% agarose gel electrophoresis, and DNA concentrations were quantified using a Qubit fluorometer. The libraries were subsequently pooled for sequencing.

Purified amplicon libraries were subjected to full-length 16S rRNA gene sequencing using the PacBio single-molecule real-time (SMRT) sequencing platform. Raw subreads were processed using the CCS module of SMRT Link software (v10.0) with the parameters -min-passes 3 -min-rq 0.999 to generate high-quality circular consensus sequence (CCS) reads. CCS reads were demultiplexed according to barcode sequences using lima with the parameters -ccs -different -peek-guess. The resulting BAM files were converted to FASTQ format using bam2fastq, and PRINSEQ (v0.20.4) was used to retain reads ranging from 1000 to 1600 bp. The software versions reported for the analysis included PRINSEQ (v0.20.4), DADA2 (v1.14.0), RDP classifier (v2.12), Mothur (v1.43.0), R (v3.6.0), and SMRT Link (v10.0).

After quality filtering, 50,000–68,000 valid sequences were retained per sample, with mean sequence lengths of approximately 1450–1490 bp. Sequence denoising, error correction, dereplication, and chimera removal were performed using the DADA2 package (v1.14.0) in R to generate amplicon sequence variants (ASVs). The DADA2 workflow generated amplicon sequence variants (ASVs) at single-nucleotide resolution without clustering sequences at the conventional 97% similarity threshold. These ASVs were used as the basic sequence features for subsequent taxonomic and ecological analyses.

Taxonomic assignment of representative sequences was performed using the RDP classifier (v2.12) against the SILVA 16S rRNA database. The RDP classifier uses a naïve Bayesian assignment algorithm to assign taxonomic identities, and bacterial community composition was summarized at the domain, phylum, class, order, family, genus, and species levels. The analysis report specifically states that 16S amplicon and full-length sequences were classified using the RDP classifier against the SILVA database. Alpha diversity was evaluated using the Chao, ACE, Shannon, Simpson, Shannon evenness, and coverage indices calculated using Mothur (v1.43.0). Rarefaction curves were generated using Mothur and visualized in R to assess whether the sequencing depth adequately represented bacterial community diversity. Beta-diversity analyses were performed to evaluate differences in bacterial community composition among samples. Principal component analysis (PCA) was conducted in R based on community composition data to visualize similarities and differences in bacterial community structure among samples; samples positioned closer to each other in the ordination space were considered to have more similar community compositions.

The sequencing data has been submitted to NCBI under accession number [PRJNA1534652].

Since microbial abundance data are compositional in nature, the relative abundance table of bacterial taxa was subjected to centered log-ratio (CLR) transformation before correlation analysis. Zero values in the abundance matrix were replaced with a small pseudocount (10^−6^) to enable logarithmic transformation. The CLR transformation was calculated for each sample according to the following equation (Equation (3)):

CLR (*x_i_*) = In (*x_i_*/*g*(*x*)) (3)

where *x_i_* represents the relative abundance of each bacterial taxon in a sample, and *g*(*x*) represents the geometric mean of all taxa abundances within the same sample. The resulting CLR-transformed bacterial abundance matrix was subsequently used for downstream correlation analysis.

### 2.6. Non-Volatile Metabolite and Lipid Extraction

Non-volatile metabolites and lipids were extracted according to Chen et al. [12,16]. Briefly, 2 g of minced fish muscle was transferred into a 50 mL centrifuge tube, followed by the addition of 2.5 mL of water and 3 mL of methanol. The mixture was homogenized at 6800 rpm for 1 min at 4 °C. Subsequently, 10 mL of methyl tert-butyl ether was added, and the mixture was centrifuged at 8000× *g* for 10 min at 4 °C to induce phase separation. The upper and lower phases were collected separately. The upper phase was evaporated to dryness under a stream of nitrogen and reconstituted in acetonitrile/isopropanol (1:1, *v*/*v*) for subsequent lipidomic analysis. The lower phase was dried using a vacuum freeze concentrator (CentriVap, Labconco Corporation, Kansas City, MO, USA) and reconstituted in methanol for subsequent non-volatile metabolite profiling.

### 2.7. Analysis of Non-Volatile Metabolites

Non-volatile metabolites were characterized by untargeted metabolomic analysis following the method of Chen et al. [12]. Chromatographic separation was achieved on an Acquity UPLC BEH Amide column (2.1 mm × 150 mm, 1.7 μm; Waters, MA, USA) fitted with an Acquity UPLC BEH Amide VanGuard pre-column (2.1 mm × 5 mm, 1.7 μm). The column temperature was maintained at 45 °C. The mobile phases consisted of water (phase A) and acetonitrile/water (95:5, *v*/*v*; phase B), both containing 10 mM ammonium formate and 0.125% formic acid. The flow rate was set at 0.4 mL/min. The gradient program was as follows: 0–2 min, 100% B; 2–7.7 min, 100–70% B; 7.7–9.5 min, 70–40% B; 9.5–10.25 min, 40–30% B; 10.25–12.75 min, 30–100% B; and 12.75–17 min, 100% B. The injection volume was 1 μL, and the autosampler temperature was maintained at 5 °C. Mass spectrometric detection was performed using a Q Exactive HF-X Orbitrap mass spectrometer (Thermo Fisher, Bremen, Germany) equipped with an electrospray ionization (ESI) source. Data were acquired in both positive and negative ionization modes over an *m*/*z* range of 60–900. Metabolite annotation was performed using MS-DIAL (v4.90) based on corrected retention time, accurate mass, and fragment-ion matching, and the annotated metabolites were assigned MSI level 2 confidence. A pooled quality-control (QC) sample was prepared by mixing equal aliquots of all study samples and was periodically injected throughout the analytical sequence to monitor analytical stability. During data preprocessing, features with a relative standard deviation (RSD) greater than 30% in the QC samples were removed. In addition, features detected in fewer than 80% of the study samples were excluded to reduce the influence of low-frequency and poorly reproducible signals. The results are expressed as peak intensity.

### 2.8. Analysis of Lipid Profile

Lipidomic analysis was performed with reference to the method described by references [17,18]. Lipid separation was achieved using an Acquity UPLC CSH C18 column (2.1 mm × 100 mm, 1.7 μm; Waters Co., Milford, MA, USA) coupled to a UPLC system (LC-30AD, Shimadzu Co., Kyoto, Japan). The mobile phase consisted of acetonitrile/water (60:40, *v*/*v*) containing 10 mM ammonium formate as solvent A and isopropanol/acetonitrile (90:10, *v*/*v*) containing 10 mM ammonium formate as solvent B. The gradient elution program was as follows: 0–2 min, 15–30% B; 2–2.5 min, 30–48% B; 2.5–11 min, 48–82% B; 11–11.5 min, 82–99% B; 11.5–12 min, 99% B; 12–12.1 min, 99–15% B; and 12.1–15 min, 15% B. The injection volume was 1 μL. Mass spectrometric analysis was performed using a TripleTOF 5600 mass spectrometer (AB SCIEX, Framingham, MA, USA) equipped with an ESI source. The acquired data were processed using MS-DIAL (v4.90) for lipid annotation based on accurate mass, retention time, and MS/MS fragmentation information, and the annotated lipid species were assigned MSI level 2 confidence. A pooled QC sample was prepared by mixing equal aliquots of all study samples and was periodically injected throughout the analytical sequence to monitor analytical stability. Blank samples were included to identify background and contaminant signals, which were removed during data preprocessing. Features with an RSD greater than 30% in the pooled QC samples were removed, and features detected in fewer than 80% of the study samples were excluded. No additional normalization of peak intensities was performed prior to statistical analysis. Peak intensities were used to represent the relative abundance of the detected lipid features rather than absolute concentrations. Analytical stability was further evaluated by PCA including the pooled QC samples.

### 2.9. Statistical Analysis

Data were presented as mean ± standard deviation (SD), with each individual fish sample considered as the experimental unit. One-way analysis of variance (ANOVA) was performed to evaluate differences among the six commercial product groups, with product group considered as the fixed factor. Tukey’s honestly significant difference (HSD) test was applied for post hoc multiple comparisons and to control the family-wise error rate associated with pairwise comparisons. Differences were considered statistically significant at *p* < 0.05. In all figures, error bars represent SD, and different lowercase letters indicate statistically significant differences among groups according to Tukey’s HSD test (*p* < 0.05).

Lipidomic and metabolomic data were processed using MS-DIAL (v4.90). PCA was used to visualize overall compositional variation, whereas partial least squares discriminant analysis (PLS-DA) and variable importance in projection (VIP) scores were used as exploratory multivariate approaches to characterize product-separation patterns and variables associated with such separation. Heatmap analyses were performed using MetaboAnalyst (v5.0), OmicStudio (https://www.omicstudio.cn/tool), and TBtools (v1.097), as appropriate. Spearman correlation analysis was performed using the mean value of each variable for each of the six product groups as an exploratory analysis to assess potential associations among volatile compounds, bacterial taxa, metabolites, and lipids. The relative abundance table of bacterial taxa was subjected to CLR transformation before correlation analysis. The *p*-values obtained from the correlation analyses were adjusted using the Benjamini–Hochberg false discovery rate (FDR) correction, and FDR-adjusted *p*-values (q-values) < 0.05 were considered statistically significant. These correlations were interpreted as associative rather than causal relationships. Other figures were prepared using GraphPad Prism (v9.5) and OriginPro (v9.0).

Volatile compound concentrations were expressed on a wet-weight basis because the objective of this study was to characterize the commercial products in their marketed and consumable state. Accordingly, the reported concentrations represent the amounts of volatile compounds present per unit mass of the products as commercially available, thereby allowing direct comparison of their flavor-related chemical composition in the actual consumption form. The metabolomic and lipidomic data were interpreted using the same rationale, with measurements based on the products in their original marketed state rather than after normalization to dry matter. However, moisture content was not determined; therefore, dry-matter normalization could not be performed, and potential differences in residual moisture or degree of dehydration among products should be considered when interpreting the observed wet-weight differences.

## 3. Results and Discussion

### 3.1. Aroma Profile of Grass Carp

**Figure 1:**
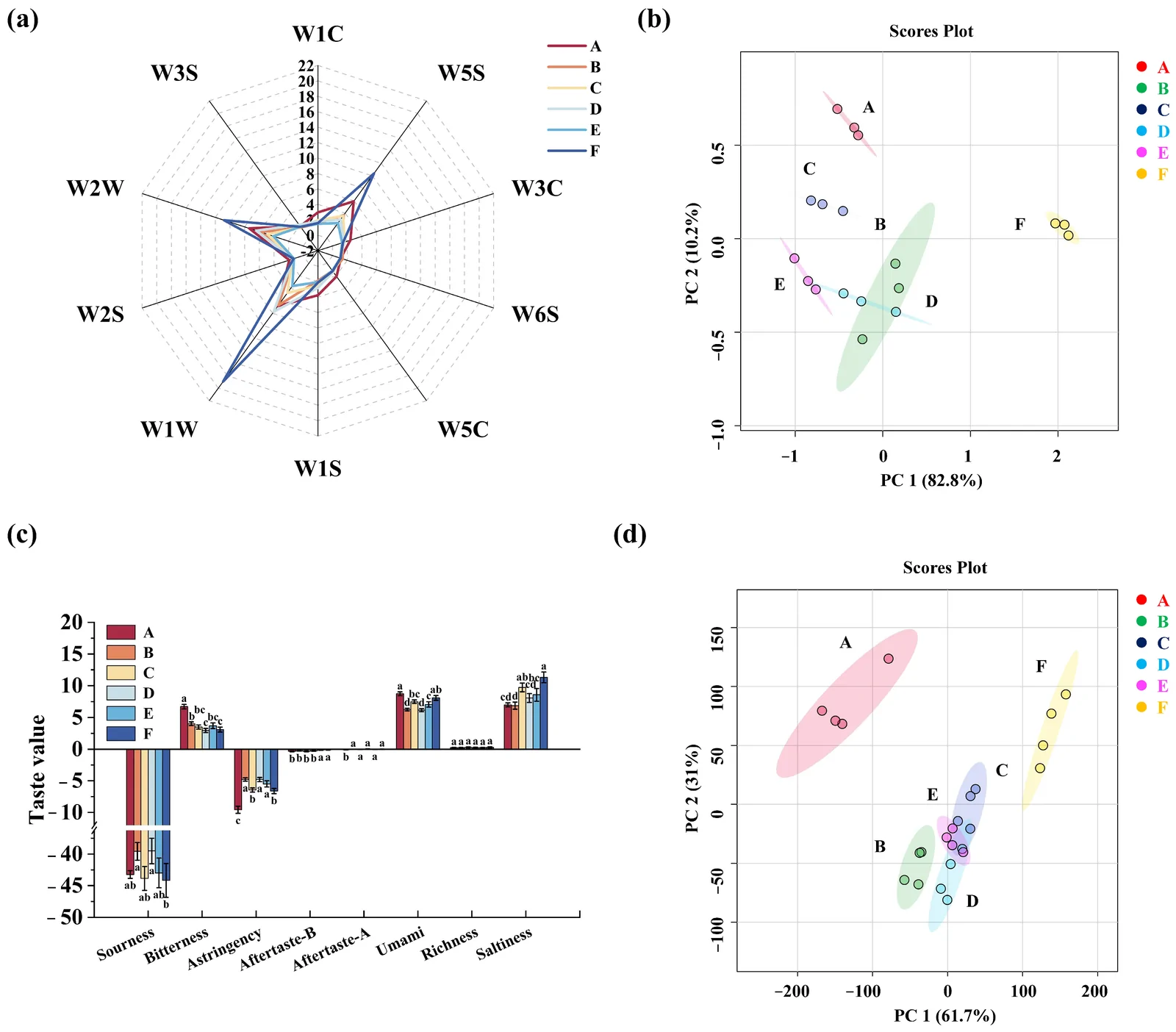
Analysis of aroma and taste profiles in grass carp. Electronic nose response signal radar plot (*n* = 3) (**a**), electronic nose PCA scores plot (**b**), electronic tongue bar chart (*n* = 4) (**c**), and electronic tongue PCA scores plot (**d**). Values of different groups with different letters (a–d) are significantly different at *p* < 0.05.

Electronic nose is an analytical instrument that mimics biological olfaction using sensor arrays and pattern recognition to rapidly identify the overall aroma of food samples [5]. Rather than directly reproducing human olfactory perception, the electronic nose generates characteristic response fingerprints that can be used to discriminate differences in volatile profiles among samples. The overall aroma profile of grass carp was shown in Figure 1a. Among all the samples, all ten sensors showed certain response values, indicating that the aroma components of grass carp are quite diverse. Among these sensors, W5S, W1W, and W2W showed relatively high response values in grass carp, indicating a strong response to nitrogen oxides and sulfur-containing compounds, which may contribute substantially to the overall aroma profile.

The main differences among the six grass carp samples were observed in the sensor response patterns and magnitudes, rather than in the presence or absence of responses from specific sensors. Specifically, Group F exhibited the highest response values for the W5S, W1W, and W2W sensors, indicating stronger sensor responses to the classes of volatile compounds to which these sensors are sensitive. The PCA results of the electronic nose data are presented in Figure 1b. The first two principal components explained 93.0% of the total variance, with PC1 and PC2 accounting for 82.8% and 10.2%, respectively. The samples exhibited a gradient-like distribution along PC1, with Groups E, C, A, B, D, and F positioned sequentially from left to right. Notably, Group F was clearly separated from the other groups in the PCA score plot, indicating a distinct electronic-nose response profile among the six sampled products.

### 3.2. Taste Profile of Grass Carp

The electronic tongue is a pattern-recognition sensor system based on artificial lipid membrane sensors, which detects electrochemical responses associated with taste-active compounds and provides comparative information on taste-related response profiles among samples [19]. Rather than directly reproducing human taste perception, the electronic tongue provides instrumental response patterns that facilitate comparison and discrimination among samples. The overall taste-related response profiles of grass carp samples are shown in Figure 1c. Among all samples, sourness showed the strongest sensor response, whereas astringency, umami, and saltiness exhibited intermediate responses. Bitterness showed relatively weak responses, and the aftertaste-related indices were low.

The sensor response values for sourness, bitterness, astringency, umami, and saltiness differed significantly among samples. Group F exhibited relatively lower sensor responses associated with bitterness and astringency, but higher responses associated with sourness and saltiness (*p* < 0.05). PCA (Figure 1d) showed that PC1 and PC2 explained 61.7% and 31.0% of the total variance, respectively, together accounting for 92.7% of the variation in the electronic tongue dataset. The distribution of samples in the PCA score plot revealed differences in their instrumental response patterns. Negative sensor values represent responses below the reference baseline of the electronic tongue system and should not be interpreted as negative taste intensities or the absence of the corresponding taste attribute. Overall, Group F exhibited a distinct electronic tongue response profile, reflecting differences in taste-related chemical characteristics among the commercial dry-cured grass carp products.

### 3.3. Volatile Composition and Content of Grass Carp

#### 3.3.1. The Overall Volatile Composition of Grass Carp

**Figure 2:**
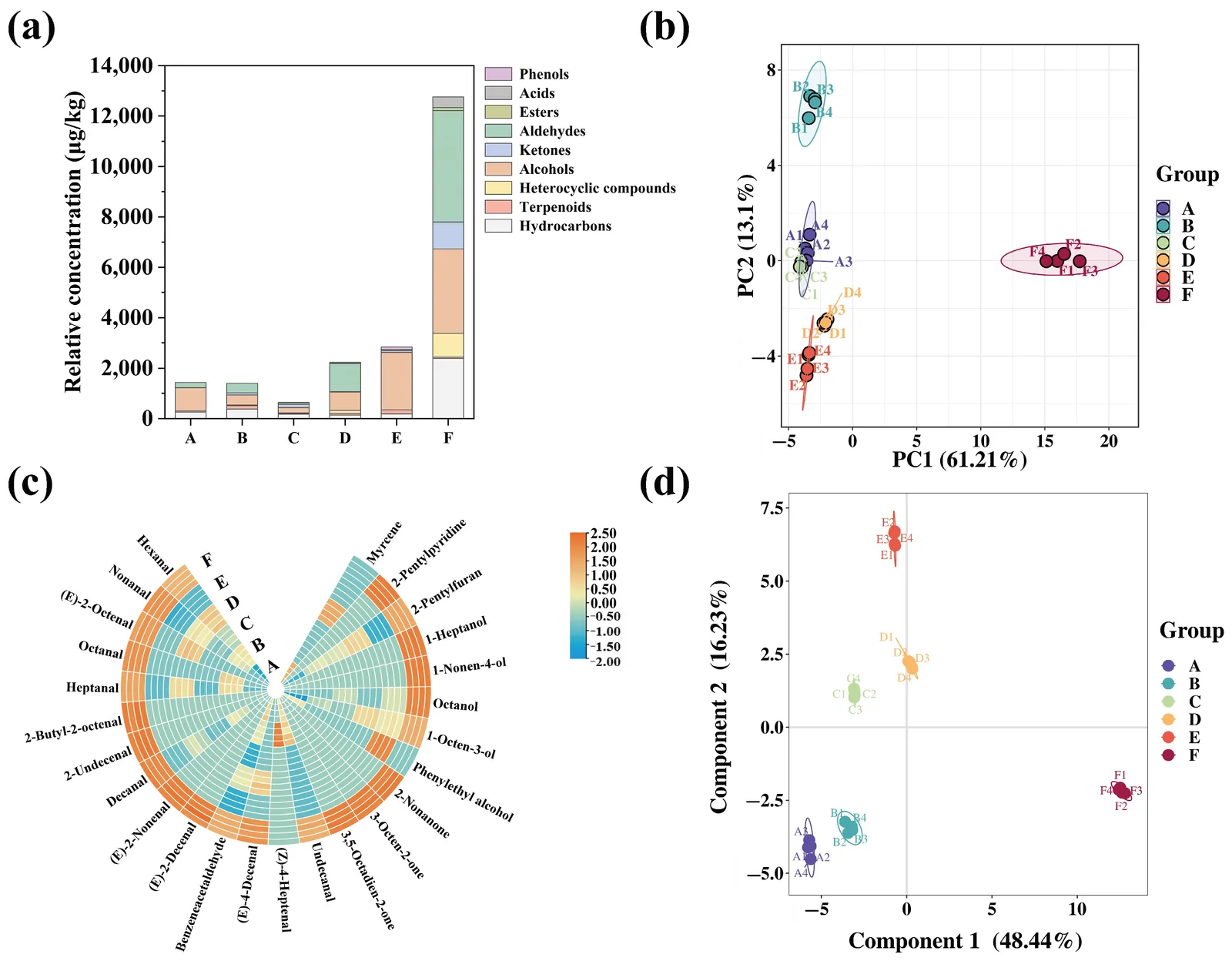
Analysis of volatile compounds in grass carp (*n* = 4). Stacked histograms of the relative content of volatile compounds (**a**), PCA scores plot (**b**), heatmap of key volatile compounds (OAV > 1) (**c**), and PLS-DA scores plot (**d**).

To better determine the compositions and contents of volatile compounds responsible for the sensory differences in grass carp, further GC-MS analysis was performed. In total, 88 volatile compounds were detected in grass carp (Table S3), comprising 19 hydrocarbons, 9 terpenoids, 4 heterocyclic compounds, 17 alcohols, 11 ketones, 17 aldehydes, 4 esters, 6 acids and 1 phenol. Groups A, B, C, D, E, and F contained 33, 38, 30, 34, 40, and 61 volatile compounds, respectively. As illustrated in Figure 2a and Table S3, alcohols, aldehydes, and hydrocarbons were present in higher amounts across all samples, whereas terpenoids, esters, and phenols were found in lower concentrations. On a wet-weight basis, Group F showed the highest measured total volatile compound content (12,762.67 μg/kg) and the greatest diversity of volatile compounds, with 61 compounds detected. Furthermore, the PCA score plot showed that Group F was located on the positive side of PC1 and exhibited a clear separation from the other groups, suggesting that Group F differed the most from the others (Figure 2b).

Aldehydes are the most important component in the flavor of grass carp. Due to their low odor thresholds, they make the greatest contribution to the overall flavor [20]. Aldehydes in grass carp primarily come from the enzymatic or auto-oxidation of unsaturated fatty acids (UFAs), which are the core compounds that contribute to the flavors, including grassy, fatty and fishy [21]. Among them, nonanal and hexanal could be detected in most of the samples and their contents were relatively high in all of them. They all belong to saturated aldehydes, among them, hexanal is formed when linoleic acid undergoes oxidation and has a pronounced grassy and fishy taste while nonanal is the product of the oxidation of oleic acid which has been described as orange scent and fatty [22]. On a wet-weight basis, the contents of nonanal and hexanal were 1391.12 μg/kg and 1441.81 μg/kg in Group F, which were much greater than those of the other groups (*p* < 0.05).

Alcohols are mainly produced during bacterial action, lipid oxidation, and protein degradation processes, and they can impart fragrances such as fishy, mush roomy, grassy, and fruity scents to the food [23]. Among them, 1-octen-3-ol was found in every sample and was present in relatively high amounts across all of them. Thus, 1-octen-3-ol was identified as an important contributor to the flavor of grass carp. This compound is primarily derived from the oxidation of UFAs, especially linoleic acid, and contributes a characteristic mushroom-like odor due to its extremely low odor threshold [24]. On a wet-weight basis, Groups A, B, C, D, E and F contained 37.69, 251.30, 173.50, 678.30, 477.38 and 1589.81 μg/kg of 1-octen-3-ol, respectively, showing that Group F had the highest concentration of 1-octen-3-ol (*p* < 0.05).

Furthermore, during the auto-oxidation process of UFAs, unstable hydroperoxides are generated [25]. These hydroperoxides further decompose to produce alkyl free radicals and then break to result in the formation of volatile hydrocarbons [25]. Hydrocarbons have a fairly high threshold, so they contribute only a small part to the flavor of grass carp [26]. However, seven types of hydrocarbons, including decane, 3-methylundecane, 3-methyltridecane, dodecane, tridecane, pentadecane and tetradecane, were all detected in the six samples. The total hydrocarbon concentration was greatest in Group F, reaching 2392.57 μg/kg. Because the products were analyzed in their marketed state and compound concentrations were expressed on a wet-weight basis, differences in residual moisture may partially contribute to the observed quantitative differences among products.

#### 3.3.2. Key and Candidate Volatile Compounds of Grass Carp Products

The OAV reflects the contribution of a volatile compound to the overall flavor, and compounds with OAVs > 1 are generally regarded as key flavor compounds [27]. The odor threshold values were obtained from reference [14], 25 volatile compounds with OAV > 1 were identified as key compounds (Table 1). In detail, 2-pentylfuran, octanol, 1-octen-3-ol, undecanal, (*E*)-4-decenal, decanal, benzeneacetaldehyde, heptanal, octanal, (*E*)-2-octenal, nonanal, and hexanal have the highest content in Group F compared with other groups (Figure 2c). Furthermore, 2-pentylpyridine, 1-heptanol, 1-nonen-4-ol, 2-nonanone, 3-octen-2-one, 3,5-octadien-2-one, (*E*)-2-decenal, (*E*)-2-nonenal, 2-undecenal, and 2-butyl-2-octenal were only detected in Group F (Figure 2c).

The PLS-DA model showed separation among the six sampled commercial products, with R^2^= 0.997 and Q^2^ = 0.965. Seven-fold cross-validation and 200-permutation testing were used to assess model performance and validation, respectively. In the permutation test, the regression intercepts for R^2^ and Q^2^ were 0.0587 and −0.6889, respectively, and the permutation-derived R^2^ and Q^2^ values were generally lower than those of the original model (Figure S1). Given the limited sample size relative to the high dimensionality of the dataset, the high R^2^ and Q^2^ values should be interpreted cautiously. VIP scores were used to rank compounds according to their contribution to group discrimination, with VIP > 1 used as a criterion for prioritizing exploratory candidate features. Accordingly, 48 volatile compounds met this criterion and were retained as exploratory candidate features (Figure S2). Of these, based on the criteria of VIP > 1 and OAV > 1, sixteen compounds—2-pentylpyridine, 1-heptanol, 1-nonen-4-ol, octanol, 1-octen-3-ol, 2-nonanone, 3-octen-2-one, 3,5-octadien-2-one, undecanal, (*E*)-4-decenal, benzeneacetaldehyde, (*E*)-2-decenal, (*E*)-2-nonenal, 2-undecenal, 2-butyl-2-octenal, and (*E*)-2-octenal were prioritized as candidate aroma-active compounds contributing to flavor differences among the sampled products. Overall, Group F exhibited the highest content and diversity of volatile compounds, identifying it as the most distinct in terms of volatile flavor profile among all grass carp samples. Although OAVs were calculated using reported odor thresholds, these thresholds are generally obtained from aqueous model systems and may not fully represent odor perception in complex food matrices. The high salt content, lipid composition, protein interactions, and matrix effects of dry-cured fish products may alter the release and sensory contribution of volatile compounds. Therefore, OAVs in the present study should be interpreted as indicators of potential aroma contribution rather than direct measurements of sensory impact. Sensory evaluation and matrix-specific odor threshold determination are required for further validation.

### 3.4. Bacterial Diversity and Composition of Grass Carp

#### 3.4.1. The Bacterial Diversity of Grass Carp

**Figure 3:**
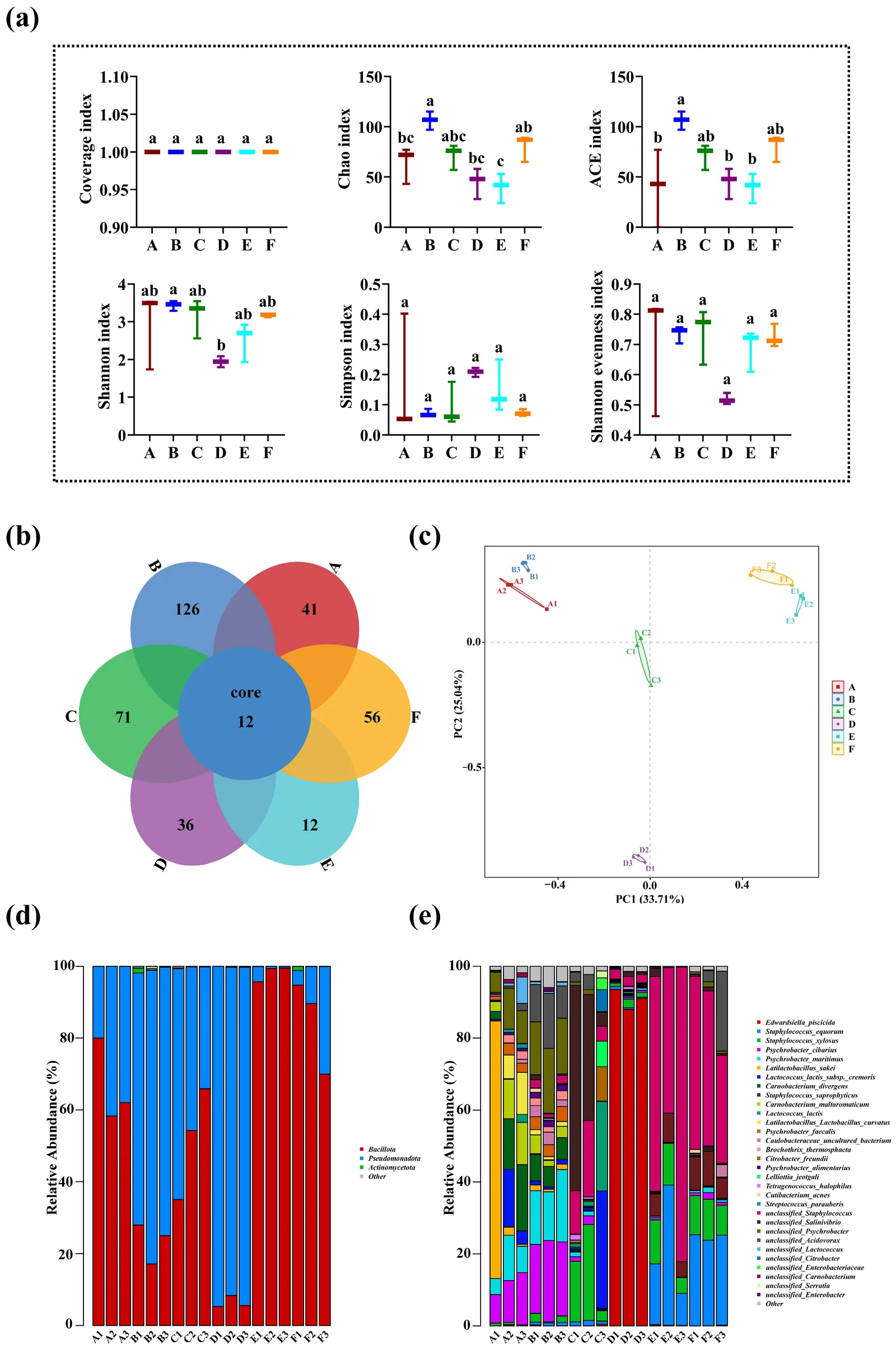
Analysis of bacterial diversity and composition in grass carp (*n* = 3). α-Diversity (**a**), Venn diagram based on the ASVs abundance of bacterial community (**b**), PCA score plot based on the ASVs’ abundance of bacterial community (**c**), relative abundance of the bacterial community at the phylum (**d**) and species (**e**) level. Values of different groups with different letters (a–c) are significantly different at *p* < 0.05.

Before diversity analysis, the quality of 16S rRNA gene amplicon sequencing was assessed. Agarose gel electrophoresis confirmed successful amplification of the target fragments (Figure S3). Rarefaction analysis demonstrated that sequencing reads were sufficient for microbial community characterization, as ASV numbers stabilized with increasing sequencing depth (Figure S4). The bacterial diversity in six grass carp samples was examined using α-diversity and β-diversity metrics (Figure 3a–c). In α-diversity analysis, Coverage indicates sequencing coverage, ACE and Chao reflect the richness of species, Shannon and Simpson indicate the diversity of species, and Shannon evenness represents the evenness of species [28,29,30]. As shown in Figure 3a, the Coverage indices of all six groups were close to 1.00 and showed no significant differences among groups, indicating sufficient sequencing depth and good coverage of the bacterial communities. The Chao and ACE indices showed differences in bacterial community richness among the groups, with Group B exhibiting the highest values, whereas Groups D and E generally showed relatively low richness indices. For the Shannon index, Group B showed the highest mean value, while Group D exhibited the lowest value. However, the Simpson index did not differ significantly among the six groups. Although the Shannon evenness index varied among groups, with higher values observed in Groups C, B, F, and E and a lower value in Group D, these differences were not statistically significant. Overall, these results indicate that bacterial community richness, diversity, and evenness varied to different extents among the six groups.

Furthermore, the flower plot results showed that the number of core ASVs common to all samples was 12 (Figure 3b). Group B had the largest number of unique ASVs (126), followed by Group C (71) and Group F (56). Subsequently, the bacterial community structures among the six grass carp products were evaluated using β-diversity analyses (Figure 3c). PCA based on ASV abundance profiles showed that the first two principal components explained 58.75% of the total variance, with distinct clustering patterns among the sampled products. Consistently, PCoA based on a Bray–Curtis dissimilarity matrix calculated from ASV abundance data (Figure S5) revealed clear clustering patterns, with PCoA1 and PCoA2 explaining 42.37% and 29.96% of the variation, respectively. Samples from Groups A and B clustered closely together, indicating similar community composition, whereas Group D was clearly separated from Groups E and F along the PCoA2 axis. PERMANOVA based on Bray–Curtis dissimilarities further indicated significant differences in bacterial community composition among the six sampled products (pseudo-F = 9.13, R^2^ = 0.79, *p* = 0.001; 999 permutations). These differences in bacterial community composition among grass carp samples may be due to the combined impact of growth status, feed nutritional composition, aquaculture water environment, and management practices [31].

#### 3.4.2. The Overall Bacterial Taxonomic Composition of Grass Carp

The overall bacterial taxonomic composition in six grass carp samples was examined at both the phylum and species levels (Figure 3d,e). At the level of phylum, a total of 3 phyla were detected with a relative abundance greater than 1%, namely *Bacillota* (*Firmicutes*), *Pseudomonadota* (*Proteobacteria*), and *Actinomycetota* (*Actinobacteria*) (Figure 3d). Among them, *Bacillota* and *Pseudomonadota* were the predominant phyla, making up about 99% of the total ASVs. Similar results were also reported in hairtail (*Trichiurus lepturus*) [32] and golden pompano (*Trachinotus blochii*) [33]. The relative abundance of *Bacillota* in the grass carp samples declined in the following sequence: Group E (98.18%) > Group F (84.78%) > Group A (66.79%) > Group C (51.73%) > Group B (23.37%) > Group D (6.38%). In contrast, the relative abundance of *Pseudomonadota* exhibited the opposite pattern.

The species-level composition of bacterial communities across different groups is shown in Figure 3e. In Group A, the bacterial community was mainly composed of *Latilactobacillus sakei* (23.57%), *Carnobacterium divergens* (11.86%), *Psychrobacter cibarius* (11.46%), and unclassified *Psychrobacter* (8.79%). Group B exhibited a predominance of *Psychrobacter cibarius* (20.81%), *Psychrobacter maritimus* (16.30%), unclassified *Psychrobacter* (16.02%), and unclassified *Acidovorax* (11.45%), suggesting a relatively high abundance of psychrotrophic bacterial populations. In Group C, unclassified *Salinivibrio* (30.92%) was the predominant taxon, followed by *Staphylococcus xylosus* (15.16%), unclassified *Staphylococcus* (12.33%), and *Lactococcus lactis* subsp. *cremoris* (12.00%). Group D showed a highly distinct bacterial profile, with *Edwardsiella piscicida* accounting for 90.74% of the bacterial community, indicating a strong enrichment of this species-level taxon. Groups E and F were characterized by the dominance of *Staphylococcus*-related taxa. In Group E, unclassified *Staphylococcus* accounted for 60.27%, followed by *Staphylococcus equorum* (21.62%) and *Staphylococcus xylosus* (9.65%). Similarly, Group F showed high abundances of unclassified *Staphylococcus* (40.46%), *Staphylococcus equorum* (24.80%), and *Staphylococcus xylosus* (10.13%). The species-level analysis further demonstrated that bacterial community composition differed substantially among groups. The enrichment of *Latilactobacillus sakei* and *Carnobacterium divergens* in Group A suggests a greater contribution of lactic acid bacteria-associated populations, whereas the high abundance of *Psychrobacter* species in Groups A and B indicates their potential adaptation to the storage environment. The predominance of *Edwardsiella piscicida* in Group D and the enrichment of *Staphylococcus*-related taxa in Groups E and F highlight different microbial succession patterns among products.

The high abundance of *Edwardsiella* and *Staphylococcus*-related taxa in some groups deserves further discussion. In this study, bacterial communities were characterized using PacBio SMRT-based full-length 16S rRNA gene sequencing, allowing improved taxonomic resolution compared with short-read amplicon sequencing. Species-level annotation indicated that the predominant taxa were mainly assigned as *Edwardsiella piscicida*, *Staphylococcus equorum*, *Staphylococcus xylosus*, and unclassified *Staphylococcus* rather than direct identification of *Staphylococcus aureus* or *Edwardsiella tarda*. It should be noted that members of the genus *Staphylococcus* are frequently detected in fermented foods. Coagulase-negative staphylococci (CNS), including *Staphylococcus xylosus* and *Staphylococcus equorum*, have been reported as important microorganisms in fermented meat products due to their contribution to proteolysis, lipolysis, aroma compound formation, and color development [34]. Similarly, *S. equorum* has been isolated from various fermented food environments and has been investigated for its potential technological functions in fermentation, including the production of flavor-related compounds and adaptation to high-salt environments [35]. Therefore, the presence of *Staphylococcus*-related taxa in the present study does not necessarily indicate poor hygiene or contamination by pathogenic bacteria.

However, species-level assignment based on 16S rRNA sequences should still be interpreted cautiously, as strain-level characteristics, including virulence factors, antibiotic resistance, and biogenic amine production capacity, cannot be determined by amplicon sequencing alone. Previous studies have demonstrated that closely related bacterial species or strains may exhibit substantially different functional and safety characteristics, and therefore genome-based analyses or targeted molecular assays are required for accurate safety evaluation. Food-derived CNS strains require further safety evaluation before application as starter cultures. If the dominant strains identified in this study are intended for isolation and development as potential fermentation starters, subsequent studies should include strain isolation, whole-genome sequencing or targeted PCR assays, evaluation of safety-related genes, antimicrobial susceptibility testing, assessment of biogenic amine production ability, and verification of fermentation performance through controlled inoculation experiments.

### 3.5. Non-Volatile Metabolite Composition of Grass Carp

#### 3.5.1. The Overall Non-Volatile Metabolite Composition of Grass Carp

**Figure 4:**
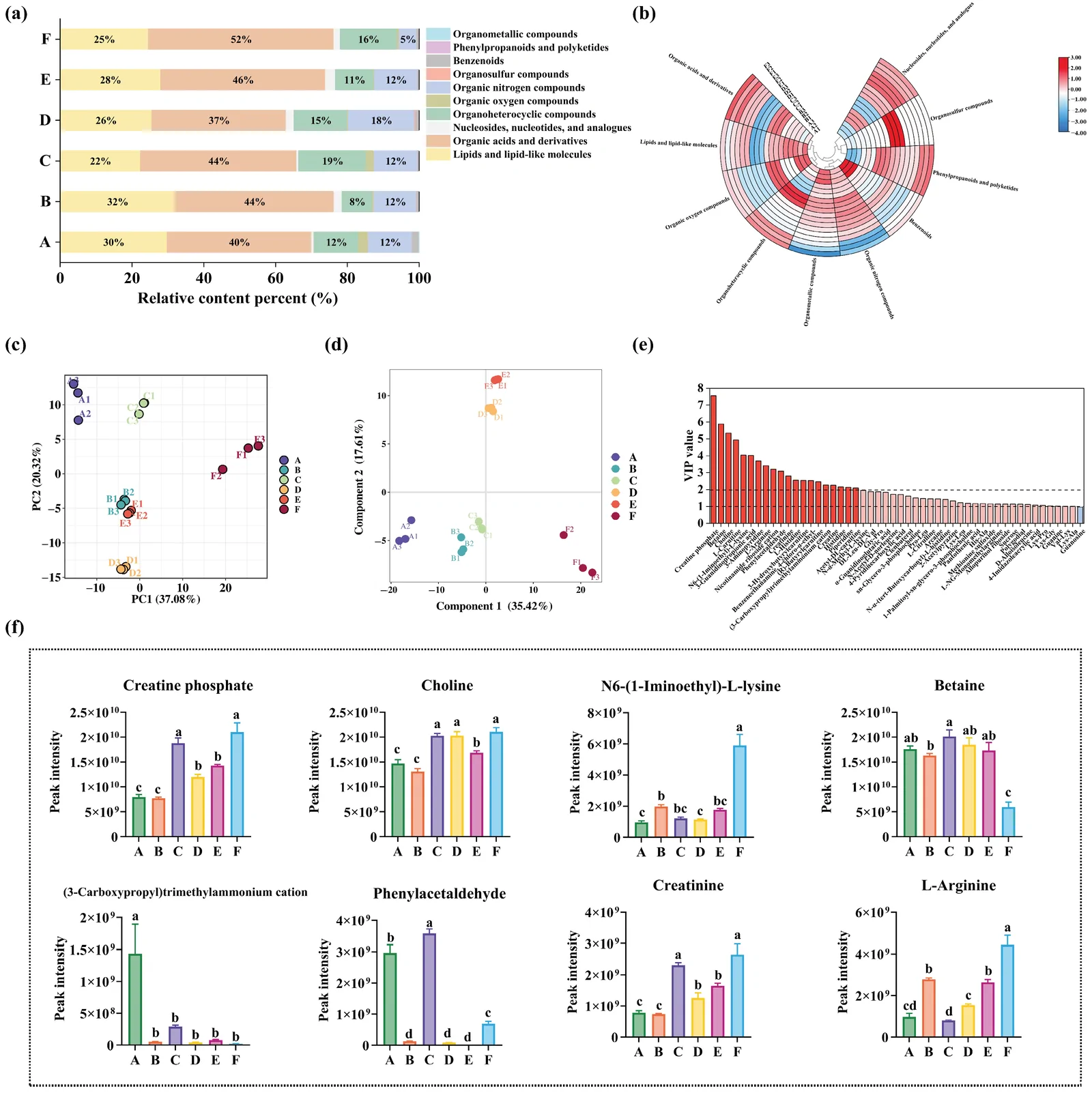
Analysis of non-volatile metabolite composition in grass carp (*n* = 3). Metabolite classes and relative content percentages (**a**), heatmap visualization of metabolite classes (**b**), PCA scores plot of metabolites (**c**), PLS-DA scores plot of metabolites (**d**), VIP scores plot of metabolites based on PLS-DA analysis (**e**), and the content of represented metabolites (VIP > 2) (**f**). Values of different groups with different letters (a–d) are significantly different at *p* < 0.05.

Using the HMDB superclass classification, 380 metabolites were identified in grass carp and grouped into 10 categories. The relative content of these metabolites is illustrated in Figure 4a. Among these, organic acids and derivatives were the most abundant (37–52%), followed by lipids and lipid-like molecules (22–32%), organoheterocyclic compounds (8–19%), and organic nitrogen compounds (5–18%). Peng et al. [36] also found that lipids and lipid-like molecules, as well as organic acids and derivatives, were the two main metabolite types in grass carp.

The heatmap showed that the metabolic characteristics varied significantly across the samples (Figure 4b and Figure S6). Specifically, the changes in organic acids and derivatives, as well as lipids and lipid-like molecules, were more pronounced, suggesting significant differences in amino acid metabolism, lipid metabolism and the composition of flavor precursor substances among different samples [37]. The QC samples (QC1, QC2, and QC3) were closely clustered near the origin of the PCA score plot (Figure S7), demonstrating the high stability and reproducibility of the analytical platform and confirming the reliability of the small-molecule metabolomics data. The PCA score plot also revealed differences in the overall small-molecule metabolite profiles among the samples (Figure 4c). On a wet-weight basis, Group F showed strong group specificity, with the highest relative signal intensities of organic acids and derivatives. They were closely related to the sourness, umami and overall flavor of the fish meat [38], suggesting that Group F may have more obvious characteristics of flavor substance accumulation. These results were consistent with those obtained from the electronic tongue analysis.

#### 3.5.2. Exploratory Candidate Metabolite Features in Grass Carp Products

PLS-DA was performed on UV-scaled data using seven-fold cross-validation to evaluate differences in metabolite profiles among the sampled groups. Model validation was further assessed using 200 permutation tests, in which class labels were randomly permuted and the corresponding R^2^ and Q^2^ values were recalculated. The original PLS-DA model yielded R^2^ = 0.987 and Q^2^ = 0.917 (Figure S8). The permutation analysis showed that the R^2^ and Q^2^ values of the original model were higher than those obtained from the permuted models, with regression intercepts of 0.1978 for R^2^ and −1.0633 for Q^2^. These results supported the observed multivariate separation; however, given the limited sample size and high dimensionality of the dataset, the PLS-DA results were interpreted primarily as exploratory (Figure 4d). Based on VIP ranking, 49 metabolites with VIP > 1 were prioritized as exploratory candidate features, including 20 metabolites with VIP > 2 (Figure 4e). Most of them belonged to the lipids and lipid-like molecules as well as organic acids and derivatives, once again indicating that they were the primary factors responsible for the variations in flavor.

Most lipids and lipid-like molecules identified in this category were related to choline metabolism, whereas most organic acids and derivatives comprised amino acids, modified amino acids, or their derivatives associated with amino acid metabolism. These findings suggest that differences in choline- and amino acid-related metabolic profiles may be associated with flavor variation among the sampled grass carp products. Choline metabolism has been reported to be associated with the formation of trimethylamine, which can contribute to fishy odors in aquatic products [39]. However, because trimethylamine and trimethylamine N-oxide were not measured in the present study, their contribution to the observed flavor differences cannot be directly inferred. Similarly, amino acid-related metabolites may potentially influence flavor through pathways such as Maillard reactions and Strecker degradation, as well as through their direct taste properties [40]. These proposed relationships are therefore considered literature-supported hypotheses rather than mechanisms demonstrated by the present data.

Figure 4f shows eight candidate metabolite features with VIP > 2, including creatine phosphate, betaine, choline, N6-(1-iminoethyl)-L-lysine, L-arginine, phenylacetaldehyde, (3-carboxypropyl)trimethylammonium cation, and creatine. They showed pronounced inter-variety variation, which may largely explain the differences in flavor profiles. Specifically, Group F showed higher peak intensities for various metabolites, such as creatine phosphate, N6-(1-iminoethyl)-L-lysine, creatinine, and L-arginine. Overall, variations in the distribution of organic acids and derivatives, as well as lipids and lipid-like molecules, were associated with flavor variation among grass carp samples, with Group F exhibiting the most distinct profile of flavor-related metabolites.

### 3.6. Lipid Profile of Grass Carp

#### 3.6.1. The Overall Lipid Composition of Grass Carp

**Figure 5:**
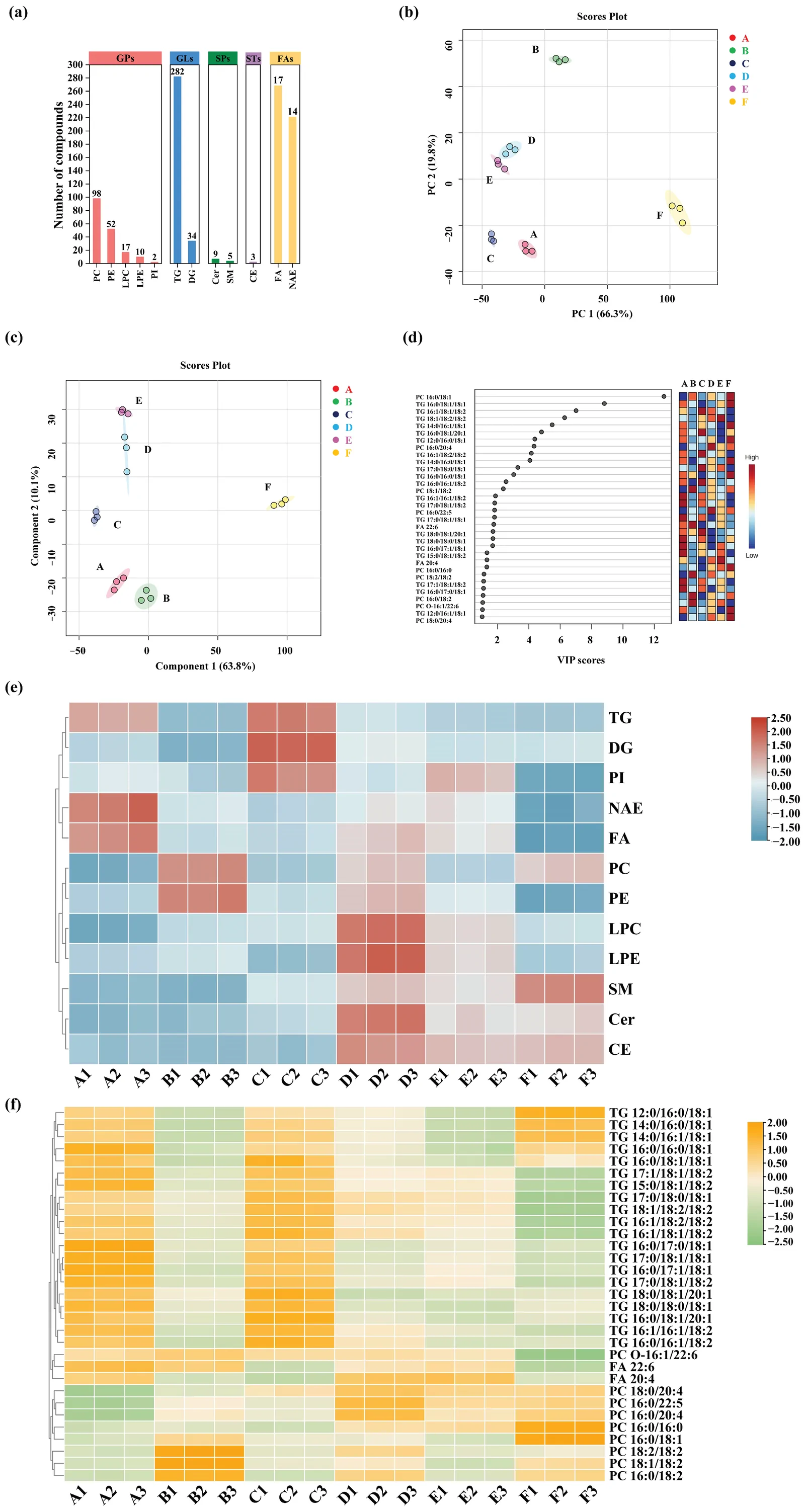
Analysis of lipid profile in grass carp (*n* = 3). The subclassification of identified lipid species (**a**), PCA scores plot of lipid species (**b**), PLS-DA scores plot of lipid species (**c**), VIP scores plot of lipid species based on PLS-DA analysis (**d**), heatmap visualization of the lipid subclasses (**e**), and heatmap visualization of represented lipid species (VIP > 1) (**f**). GPs, glycerophospholipids. GLs, glycerolipids. SPs, sphingolipids. STs, sterol lipids. FAs, fatty acyls. PC, phosphatidylcholine. PE, phosphatidylethanolamine. LPC, lyso-phosphatidylcholine. LPE, lyso-phosphatidylethanolamine. PI, phosphatidylinositol. TG, triacylglycerol. DG, diacylglycerol. Cer, ceramide. SM, sphingomyelin. CE, cholesteryl ester. FA, fatty acid. NAE, N-acylethanolamine.

The generation of flavor is strongly associated with its lipid composition [41]. As shown in Figure 5a, 543 lipid molecules detected in grass carp were mainly concentrated in two major categories: glycerolipids (GLs) and glycerophospholipids (GPs). The quantities of sphingolipids (SPs), sterol lipids (STs), and fatty acyls (FAs)-type lipid molecules were relatively low. This finding aligns with previous studies reporting that GLs and GPs are the main components of grass carp [21,42]. Among them, the GLs-type lipid molecules were the most numerous, including 282 triacylglycerols and 34 diacylglycerols. The GPs-type lipid molecules had the richest variety, including 98 phosphatidylcholines (PCs), 52 phosphatidylethanolamines (PEs), 17 lyso-phosphatidylcholines, 10 lyso-phosphatidylethanolamines and 2 phosphatidylinositols (PIs). In addition, the FAs-type lipid molecules contained 17 fatty acids and 14 N-acylethanolamines (NAEs); the SPs-type lipid molecules included 9 ceramides and 5 sphingomyelins (SMs); and the STs-type lipid molecules only had 3 cholesteryl esters (CEs). Figure S9 shows the differences in these lipid molecules among the samples.

The QC samples (QC1, QC2, and QC3) were closely clustered near the origin of the PCA score plot (Figure S10), demonstrating the high stability and reproducibility of the analytical platform and confirming the reliability of the lipidomics data. PCA was performed to evaluate overall differences in lipid composition among groups (Figure 5b). The six groups (A–F) showed clear separation, indicating marked differences in their overall lipid profiles. Group F was located farthest along the positive direction of PC1 and was clearly separated from the other groups, suggesting that it possessed a distinctive lipid composition. The heatmap (Figure 5e) further showed differences in lipid-class abundance among the six groups. In Group F, relative signal intensities of SM, PC, and CE were relatively high, whereas the levels of PI, NAE, fatty acid, and PE were relatively low. Free fatty acids, particularly unsaturated free fatty acids, and phospholipids, such as PE, are susceptible to oxidation and can serve as important precursors for volatile flavor compounds, including aldehydes, ketones, and alcohols [43]. These compounds may contribute to grassy, fatty, and other characteristic aroma attributes, although excessive lipid oxidation can also generate undesirable odors. The lipid pattern observed in Group F therefore indicates a distinctive lipid substrate composition rather than necessarily greater oxidative stability. Notably, several lipid oxidation-associated volatile compounds, including hexanal, nonanal, and 1-octen-3-ol, also showed relatively high signal intensities in Group F. Because direct indices of lipid oxidation, such as peroxide value (PV), thiobarbituric acid reactive substances (TBARS), or conjugated dienes, were not determined in the present study, the degree of lipid oxidation in Group F cannot be inferred solely from lipid-class abundance.

#### 3.6.2. Exploratory Candidate Lipid Features in Grass Carp

To further explore lipid features contributing to differences among the groups, PLS-DA was performed (Figure 5c). PLS-DA was performed using a three-component model, with the number of components selected based on five-fold cross-validation. The final model yielded cumulative R^2^ = 0.977 and Q^2^ = 0.941 (Figure S11), indicating a high degree of fit and cross-validated predictive performance. Given the limited sample size and high dimensionality of the dataset, these PLS-DA results were interpreted as exploratory and were used primarily to characterize multivariate differences among the sampled products. There were a total of 32 lipid molecules with VIP > 1, including PC 16:0/18:1, TG 16:0/18:1/18:1, TG 16:1/18:1/18:2, TG 18:1/18:2/18:2, TG 14:0/16:1/18:1, TG 16:0/18:1/20:1, TG 12:0/16:0/18:1, PC 16:0/20:4, TG 16:1/18:2/18:2, TG 14:0/16:0/18:1, TG 17:0/18:0/18:1, TG 16:0/16:0/18:1, TG 16:0/16:1/18:2, etc. (Figure 5d). Among them, on a wet-weight basis, most of the PC molecules were significantly enriched in Group F, especially PC 16:0/16:0 and PC 16:0/18:1 (Figure 5f). However, this compositional feature should not be interpreted as evidence of greater phospholipid oxidative stability. Overall, the unique lipid profile of Group F, characterized by higher relative peak intensities of SM, PC, and CE but lower relative peak intensities of fatty acids and PE on a wet-weight basis, indicates distinct lipid substrate characteristics among the groups. These lipid compositional differences may contribute to variations in the formation potential of lipid-derived flavor compounds; however, their direct effects on lipid oxidation and sensory attributes require further validation.

### 3.7. Correlation Analysis and Potential Associations Linking Volatiles, Microorganisms, Lipids, and Metabolites

**Figure 6:**
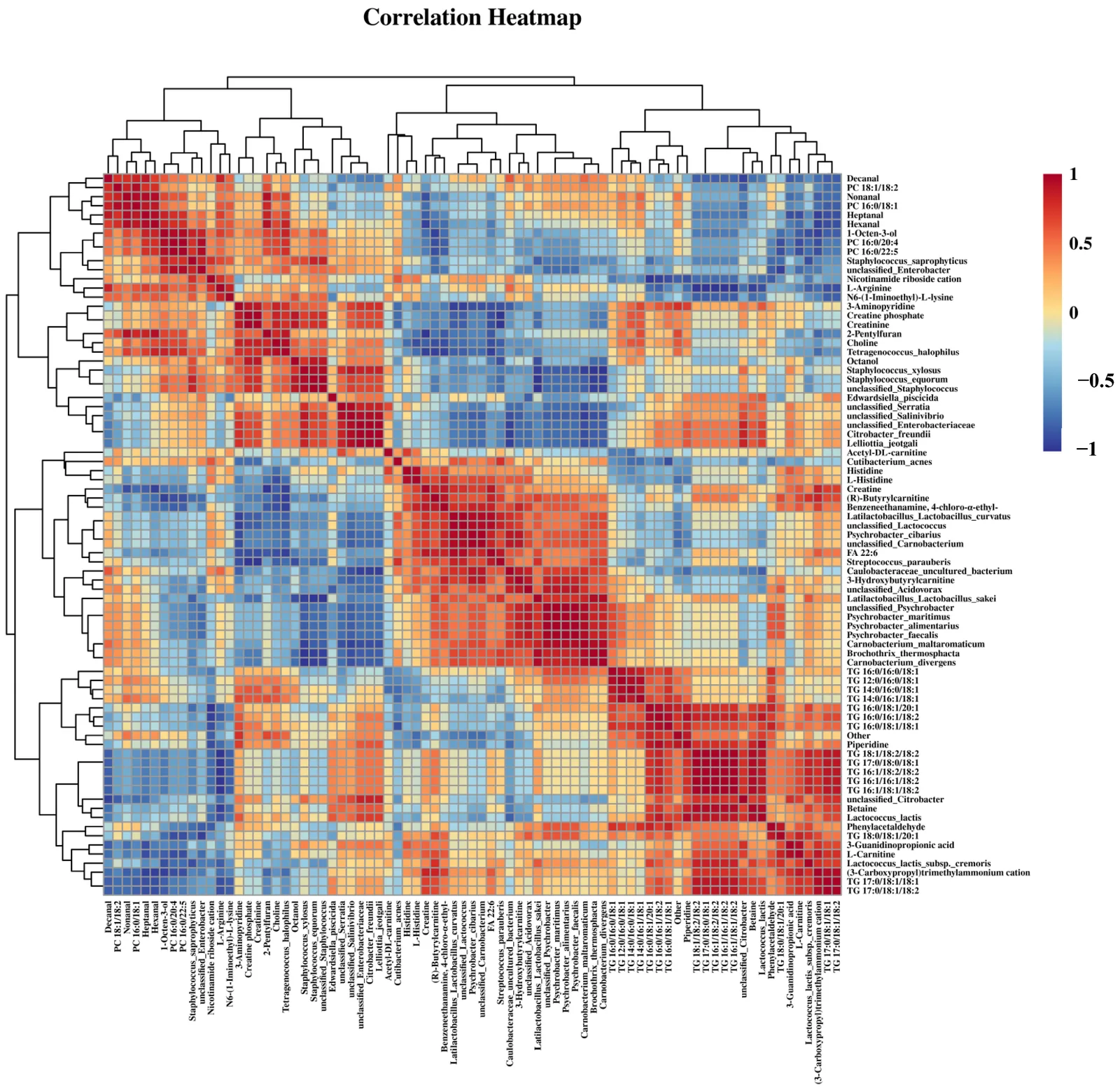
Spearman correlation analysis between CLR-transformed bacterial taxa and flavor-related compounds.

To explore potential product-level relationships between microbial communities and flavor-related characteristics, Spearman correlation analysis was performed between CLR-transformed bacterial abundance data and flavor-related chemical components, including lipids, non-volatile metabolites, and volatile compounds (Figure 6). For each variable, the mean value for each of the six commercial product groups was used; therefore, the correlation analysis was based on six product-level observations (*n* = 6), rather than individually matched biological samples. Accordingly, these correlations were interpreted as exploratory product-level associations. The CLR transformation was applied to minimize bias arising from the compositional nature of microbiome relative abundance data [44,45]. The Benjamini–Hochberg false discovery rate (FDR) correction was applied across the multiple correlation tests, with q < 0.05 considered statistically significant.

*Staphylococcus*-related taxa showed an exploratory positive association with octanol (ρ = 0.880, q = 0.388). Similarly, exploratory product-level associations were observed between lipid and volatile profiles. PC 16:0/18:1 was positively associated with nonanal (ρ = 0.943, q = 0.339), whereas several TG species showed negative associations with aldehydes, including TG 17:0/18:1/18:2 with heptanal (ρ = −0.880, q = 0.388). None of these associations remained statistically significant after FDR correction, and they were therefore considered exploratory product-level trends.

These exploratory trends may be considered in the context of previously reported biochemical pathways. Microorganisms involved in cured and fermented food systems have been reported to participate in proteolytic and lipolytic processes that can affect the availability of amino acids, free fatty acids, and other flavor precursors [9,46]. Lipid oxidation is also a well-established route for the formation of aldehydes, alcohols, and other volatile compounds from unsaturated fatty acids during processing and storage [47]. For example, hexanal and nonanal have been associated with the oxidation of linoleic acid and oleic acid, respectively [47], while nitrogen-containing metabolites derived from protein degradation may participate in Strecker degradation and contribute to the formation of aroma-active compounds [48]. These literature-supported pathways provide plausible hypotheses for interpreting the observed product-level patterns; however, the present data do not establish precursor–product relationships or causal microbial contributions to flavor formation.

An important limitation is the small number of product-level observations and the absence of individually matched cross-omics measurements. Together with the large number of simultaneous comparisons, these factors may have limited statistical power. The observed associations should therefore be regarded as hypothesis-generating patterns that require validation using larger numbers of independently sampled products and individually matched multi-omics measurements. Functional approaches, including metagenomic/metatranscriptomic analysis, microbial isolation, and enzyme activity measurements, would further help evaluate the proposed microbial contributions to flavor formation. In addition, baseline physicochemical and lipid-oxidation parameters, including moisture content, water activity, NaCl content, pH, total lipid content, PV, and TBARS, were not determined concurrently with the multi-omics analyses; therefore, their potential contributions to the observed microbial, lipid, and flavor-related variation could not be independently evaluated.

## 4. Conclusions

Integrated flavor and multi-omics analyses revealed substantial differences among the six commercial dry-cured grass carp products examined in this study. Group F was distinguished by its aroma and taste profiles, a greater diversity and higher measured levels of volatile compounds on a wet-weight basis, and distinct metabolite and lipid profiles. Species-level bacterial profiles also differed markedly among the six products, with *Edwardsiella piscicida* predominating in Group D and *Staphylococcus equorum* and *Staphylococcus xylosus* abundant in Groups E and F. Variation in metabolite profiles was particularly evident in organic acid-, amino acid-, and choline-related metabolites, whereas lipid profiles differed mainly in GLs and GPs, with Group F showing a distinctive PC-enriched pattern. Exploratory product-level correlation analysis suggested potential associations among bacterial taxa, lipid and metabolite profiles, and aroma-active volatiles; however, none of these associations remained statistically significant after FDR correction. Collectively, these findings indicate that flavor variation among the six commercial dry-cured grass carp products examined in this study was accompanied by differences in bacterial community structure and metabolite and lipid profiles, providing candidate compositional features and hypotheses for subsequent validation.

## Figures and Tables

**Table 1:** Volatile compounds with OAV > 1.00 in at least one commercial product.

Compounds	Odor Threshold (μg/kg)	Odor Characteristic	OAV					
			A	B	C	D	E	F
Myrcene	13	Sweet, spicy	1.51	-	-	1.80	-	-
2-Pentylpyridine	0.6	Fatty, grassy	-	-	-	-	-	53.05
2-Pentylfuran	6	Grassy, sweaty, green	-	4.29	4.32	19.16	-	134.97
1-Heptanol	94	Fatty, citrus	-	-	-	-	-	2.36
1-Nonen-4-ol	25	Fatty, coconut	-	-	-	-	-	11.20
Octanol	130	Fatty, citrus, rose	-	-	0.02	-	0.03	3.15
1-Octen-3-ol	1	Mushroom-like	37.69	251.30	173.50	678.30	477.38	1589.81
Phenylethyl alcohol	140	Rose, spicy	0.11	-	-	-	11.92	-
2-Nonanone	5	Fruity, sweet	-	-	-	-	-	5.48
3-Octen-2-one	20	Herbal, earthy	-	-	-	-	-	5.49
3,5-Octadien-2-one	100	Fruity, green, grassy	-	-	-	-	-	3.99
Undecanal	5	Fatty, sweet	0.73	1.04	-	-	-	1.94
(*Z*)-4-heptenal	0.8	Fatty, grassy	-	17.69	-	-	-	-
(*E*)-4-decenal	2.5	Citrus, fatty	-	-	-	1.65	-	5.78
Benzeneacetaldehyde	4	Nutty	11.22	3.85	-	3.50	-	36.90
(*E*)-2-decenal	0.3	Floral	-	-	-	-	-	165.52
(*E*)-2-nonenal	0.1	Green, grassy	-	-	-	-	-	607.34
Decanal	0.1	Fatty, warmed-over	30.68	75.80	17.02	57.16	18.91	815.39
2-Undecenal	0.7	Wax, citrus	-	-	-	-	-	173.87
2-Butyl-2-octenal	20	Fruity, sweaty	-	-	-	-	-	5.89
Heptanal	3	Fatty, grassy	-	7.32	-	8.02	-	57.62
Octanal	0.7	Fatty, soap, lemon	-	23.67	-	-	-	291.97
(*E*)-2-octenal	3	Fatty, nutty	-	-	-	12.18	-	71.32
Nonanal	1	Grassy	24.10	95.66	29.69	133.16	11.50	1391.12
Hexanal	4.5	Green, grassy	-	32.70	7.34	127.52	0.64	320.40

Note: -, not detected.

## Data Availability

The original contributions presented in this study are included in the article/Supplementary Materials. Further inquiries can be directed to the corresponding authors.

## Supplementary Materials

The following supporting information can be downloaded at: https://www.mdpi.com/article/10.3390/foods15193547/s1, Figure S1: R^2^ and Q^2^ of PLS-DA analysis of volatile compounds; Figure S2: VIP score plots of volatile compounds (top 48) based on PLS-DA analysis; Figure S3: agarose gel electrophoresis image; Figure S4: the change in ASV numbers with the sequencing depth; Figure S5: PCoA analysis based on microbial community distances; Figure S6: heatmap visualization of 380 metabolites in grass carp; Figure S7: PCA scores plot of metabolites between grass carp samples and QC samples; Figure S8: R^2^ and Q^2^ of PLS-DA analysis of non-volatile metabolites; Figure S9: heatmap visualization of 543 lipid molecules in grass carp; Figure S10: PCA scores plot of lipid molecules between grass carp samples and QC samples; Figure S11: R^2^ and Q^2^ of PLS-DA analysis of lipid profile; Table S1: allocation of individual fish samples from each commercial product across the analytical platforms; Table S2: electronic nose sensor-specific detection biases; Table S3: compositions and concentrations of volatile compounds in grass carp (μg/kg).

## Abbreviations

The following abbreviations are used in this manuscript:

UFA	Unsaturated fatty acid
GC-MS	Gas chromatography-mass spectrometry
GC-IMS	Gas chromatography-ion mobility spectrometry
OAV	Odor activity value
UPLC-MS	Ultra-performance liquid chromatography coupled with mass spectrometry
HS-SPME	Headspace solid-phase microextraction
RIs	Retention indices
SMRT	Single-molecule real-time
CCS	Circular consensus sequences
ASV	Amplicon sequence variant
PCA	Principal component analysis
CLR	Centered log-ratio
ESI	Electrospray ionization
QC	Quality-control
RSD	Relative standard deviation
SD	Standard deviation
ANOVA	Analysis of variance
HSD	Honestly significant difference
PLS-DA	Partial least squares discriminant analysis
VIP	Variable importance in projection
CNS	Coagulase-negative staphylococci
PV	Peroxide value
TBARS	Thiobarbituric acid reactive substances
FDR	False discovery rate
GL	Glycerolipid
GP	Glycerophospholipid
SP	Sphingolipid
ST	Sterol lipid
FA	Fatty acyl
PC	Phosphatidylcholine
PE	Phosphatidylethanolamine
PI	Phosphatidylinositol
NAE	N-acylethanolamine
SM	Sphingomyelin
CE	Cholesteryl ester
