# Supplementary material for: Integrated Multi-Omics Analysis Reveals Associations Between Flavor Variation, Bacterial Communities, Metabolites, and Lipids in Commercial Dry-Cured Grass Carp
Source: Foods. 2026 Oct 4;15(19):3547. doi: 10.3390/foods15193547 (PMC13664989; doi:10.3390/foods15193547)
Supplement: Supplementary file 1 [file foods-15-03547-s001.zip › foods-4578460-supplementary.pdf]

Supplementary Data

Integrated Multi-Omics Analysis Reveals Associations Between Flavor Variation, Bacterial Communities, Metabolites, and Lipids in Commercial Dry-Cured Grass Carp

Table S1. Allocation of individual fish samples from each commercial product across the analytical platforms.

Product	Fish ID	Electronic nose	Electronic tongue	GC–MS	Microbiome sequencing	Metabolomics	Lipidomics
A	A1	√	√	√	√	√	√
A	A2	√	√	√	√	√	√
A	A3	√	√	√	√	√	√
A	A4	-	√	√	-	-	-
A	A5	-	-	-	-	-	-
B	B1	√	√	√	√	√	√
B	B2	√	√	√	√	√	√
B	B3	√	√	√	√	√	√
B	B4	-	√	√	-	-	-
B	B5	-	-	-	-	-	-
C	C1	√	√	√	√	√	√
C	C2	√	√	√	√	√	√
C	C3	√	√	√	√	√	√
C	C4	-	√	√	-	-	-
C	C5	-	-	-	-	-	-
D	D1	√	√	√	√	√	√
D	D2	√	√	√	√	√	√
D	D3	√	√	√	√	√	√
D	D4	-	√	√	-	-	-
D	D5	-	-	-	-	-	-
E	E1	√	√	√	√	√	√
E	E2	√	√	√	√	√	√

E	E3	√	√	√	√	√	√
E	E4	-	√	√	-	-	-
E	E5	-	-	-	-	-	-
F	F1	√	√	√	√	√	√
F	F2	√	√	√	√	√	√
F	F3	√	√	√	√	√	√
F	F4	-	√	√	-	-	-
F	F5	-	-	-	-	-	-

Note: Each product comprised five individually packaged fish, designated A1-A5 through F1-F5. A check mark (√) indicates that the corresponding individual fish was used for the indicated analytical platform, whereas an en dash (-) indicates that the fish was not used for that analysis. Samples from individual fish were analyzed separately. No samples were pooled for microbiome sequencing, metabolomics, or lipidomics analyses. Information sufficient to verify whether the five packages within each commercial product originated from different production lots/batches was unavailable; therefore, production-batch independence could not be established.

Table S2. Electronic nose sensor-specific detection biases.

Sensors number	Sensor	Performance description
1	W1C	Aromatic organic compounds
2	W5S	Nitrogen oxides
3	W3C	Ammonia and aromatic compounds
4	W6S	Hydrides
5	W5C	Alkanes and aromatic compounds
6	W1S	Methane
7	W1W	Sulfides and terpenes
8	W2S	Alcohols, aldehydes, and ketones
9	W2W	Aromatic ingredients and organic sulfides
10	W3S	Long-chain alkanes

Table S3. Compositions and concentrations of volatile compounds in grass carp (μg/kg).

Compounds	RI[1]	RI[2]	A	B	C	D	E	F
Hydrocarbons (19)								
2,3,7-Trimethyldecane	1463	1466	2.92±0.37bc	ND	5.32±1.04a	2.28±0.81c	4.12±0.33ab	ND
Undecane	1102	1100	ND	5.02±0.41b	ND	ND	ND	6.55±0.38a
3-Methylpentadecane	1571	1570	2.96±0.91b	1.64±0.49b	ND	ND	1.7±0.16b	5.54±1.19a
5-Ethyldecane	1146	1139	6.53±1.37a	ND	ND	ND	ND	ND
4,6-Dimethyldodecane	1328	1325	1.74±0.28c	4.70±0.74b	1.43±0.20c	ND	7.05±1.21a	ND
4-Methyldecane	1062	1060	7.58±1.74ab	9.66±1.86a	6.22±0.31b	ND	ND	ND
5-Methyldecane	1059	1057	9.28±1.28a	10.46±1.59a	6.29±0.62b	ND	6.12±1.08b	ND
2-Methyldecane	1066	1064	11.18±2.48a	11.86±2.75a	6.47±0.90b	ND	3.11±0.08b	ND
Decane	1001	1000	9.78±0.87b	7.98±1.70bc	5.85±1.08c	8.22±1.21bc	6.27±0.79c	14.91±1.77a
3-Methylundecane	1172	1170	12.97±3.23b	12.79±2.01b	4.38±0.41c	5.59±0.80c	6.29±1.15c	17.96±1.22a
2,5-Dimethyldecane	1119	1112	ND	14.75±4.27a	ND	ND	ND	ND
3-Methyldecane	1072	1071	13.87±3.15b	26.64±2.7a	13.22±0.74b	ND	ND	ND
3-Methyltridecane	1371	1371	14.76±3.35b	13.84±2.81b	8.35±1.02b	9.26±2.04b	10.79±2.63b	41.59±4.83a
Hexadecane	1600	1600	25.31±3.95b	9.52±1.66c	6.22±1.91cd	ND	7.29±0.48cd	49.23±7.09a

Heptadecane	1700	1700	6.84±2.07b	46.93±15.04a	ND	4.20±0.10b	8.13±0.58b	16.14±1.91b
Dodecane	1201	1200	47.46±6.95b	49.36±4.50b	31.27±2.09c	45.17±3.30b	45.96±2.69b	71.04±4.77a
Tridecane	1301	1300	6.32±0.47c	31.96±9.97b	14.41±1.85c	10.80±0.66c	10.56±0.98c	153.00±13.75a
Pentadecane	1500	1500	38.76±8.42bc	71.24±20.8b	25.26±4.48c	19.36±3.86c	28.86±3.27c	290.10±36.48a
Tetradecane	1400	1400	41.02±5.21b	47.33±10.13b	42.73±2.64b	28.30±3.02b	38.80±3.91b	1726.51±176.43a
**Total**			**259.28±30.39bc**	**375.68±55.02b**	**177.42±9.80bc**	**133.18±10.54c**	**185.05±12.00bc**	**2392.57±217.12a**
Terpenoids (9)								
Pinene	931	933	ND	ND	ND	ND	ND	7.60±0.70a
E-Caryophyllene	1424	1424	2.45±0.20c	ND	ND	5.29±0.31b	10.11±1.58a	ND
Cymene	1025	1025	ND	ND	5.82±0.48c	4.96±0.51c	7.24±0.90b	10.63±1.00a
(*E*)-Calamenene	1528	1527	ND	8.99±2.96b	2.22±0.40c	8.14±0.73b	11.04±0.95b	18.61±2.71a
Limonene	1029	1030	23.23±1.76a	21.72±2.61a	5.12±0.45c	24.53±1.27a	14.12±2.30b	12.42±2.76b
Myrcene	992	991	19.66±1.32b	ND	ND	23.36±3.11a	ND	ND
3,5,5-Trimethyl-2-hexene	975	985	ND	29.90±5.66a	4.67±0.97c	11.51±1.4b	15.62±2.72b	ND
D-Camphor	1146	1143	ND	ND	ND	ND	74.57±2.38a	ND
3,4-Dimethoxytoluene	1242	1233	ND	71.68±16.82a	ND	ND	30.01±2.63b	ND
**Total**			**45.34±1.82d**	**132.29±20.56b**	**17.83±0.89e**	**77.79±5.04c**	**162.71±8.63a**	**49.26±4.37d**

Heterocyclic compounds(4)									
2-Ethylpyridine	908	906	ND	ND	ND	ND	ND	8.21±2.12a
2-Pentylpyridine	1200	1202	ND	ND	ND	ND	ND	31.83±2.78a
2-Octylfuran	1296	1297	ND	ND	ND	ND	ND	84.56±11.56a
2-Pentylfuran	993	993	ND	25.76±8.28c	25.89±4.18c	114.94±22.23b	ND	809.84±77.06a
**Total**			**ND**	**25.76±8.28b**	**25.89±4.18b**	**114.94±22.22b**	**ND**	**934.44±86.34a**
Alcohols (17)								
1-Nonanol	1175	1173	2.31±0.21b	ND	ND	ND	2.72±0.5b	9.47±0.70a
Benzyl alcohol	1038	1040	ND	ND	ND	ND	11.10±3.66b	19.48±0.41a
Eucalyptol	1031	1032	18.69±5.30a	ND	ND	ND	15.22±1.04a	ND
L-α-Terpineol	1193	1190	ND	21.81±4.06a	ND	ND	25.19±3.66a	ND
2-Ethyl-1-hexanol	1031	1030	ND	23.18±8.17b	8.86±2.10c	3.92±0.82c	ND	64.76±8.45a
(2*Z*)-2-Octene-1-ol	1071	1067	ND	ND	ND	39.33±6.62b	65.91±17.27a	81.16±3.19a
(*E*)-2-Decen-1-ol	1284	1270	ND	ND	ND	ND	ND	103.28±7.50a
1,3-Butanediol	787	787	ND	103.18±27.38a	ND	ND	ND	ND
Linalool	1102	1099	126.46±6.76a	ND	ND	ND	ND	ND
Hexanol	868	867	6.29±1.45c	4.85±1.49c	24.03±1.70b	2.94±0.92c	5.04±1.47c	225.38±13.44a

1-Heptanol	972	970	ND	ND	ND	ND	ND	221.79±21.52a
1-Nonen-4-ol	1097	1103	ND	ND	ND	ND	ND	280.02±29.80a
Pentyl alcohol	751	759	ND	ND	5.10±1.60b	7.35±1.88b	ND	350.10±56.65a
Octanol	1074	1076	ND	ND	2.77±0.32b	ND	3.65±0.65b	409.19±32.08a
Eugenol	1361	1357	712.61±161.46a	ND	ND	ND	ND	ND
1-Octen-3-ol	981	980	37.69±3.46e	251.30±38.31d	173.50±13.41d	678.30±34.63b	477.38±66.05c	1589.81±84.86a
Phenylethyl alcohol	1118	1116	15.38±4.29b	ND	ND	ND	1668.74±371.71a	ND
**Total**			**919.43±160.92c**	**404.32±41.54de**	**214.26±13.90e**	**731.84±39.54cd**	**2274.95±443.91b**	**3354.44±125.72a**
Ketones (11)								
Octan-3-one	988	986	7.78±0.79a	ND	ND	8.73±1.13a	6.14±0.96b	ND
Acetophenone	1068	1068	ND	ND	ND	ND	14.84±0.50b	28.64±2.72a
2-Nonanone	1094	1092	ND	ND	ND	ND	ND	27.40±2.88a
m-Ethylacetophenone	1199	1186	ND	ND	ND	ND	36.29±7.25a	ND
2,3-Octanedione	987	984	ND	ND	ND	ND	ND	58.24±3.29a
Heptalactone	1157	1155	ND	ND	ND	ND	ND	61.12±3.52a
2-Heptanone	888	891	ND	ND	ND	ND	ND	104.94±9.58a
3-Octen-2-one	1041	1040	ND	ND	ND	ND	ND	109.88±19.41a

2-Methyl-3-octanone	986	985	ND	85.93±3.16b	124.72±27.67a	ND	12.16±2.11c	ND
Nonalactone	1366	1362	ND	ND	ND	ND	ND	274.21±31.73a
3,5-Octadien-2-one	1095	1091	ND	ND	ND	ND	ND	398.88±61.98a
**Total**			**7.78±0.79c**	**85.93±3.16bc**	**124.72±27.67b**	**8.73±1.13c**	**69.43±7.62bc**	**1063.31±113.25a**
Aldehydes (17)								
Undecanal	1310	1309	3.65±1.05b	5.20±1.71b	ND	ND	ND	9.72±1.06a
(*Z*)-4-Heptenal	900	900	ND	14.16±1.30a	ND	ND	ND	ND
(*E*)-4-Decenal	1195	1198	ND	ND	ND	4.11±0.92b	ND	14.45±1.74a
3-Ethylbenzaldehyde	1166	1168	ND	29.20±6.70a	ND	ND	ND	20.48±0.59b
Benzeneacetaldehyde	1047	1045	44.88±13.53b	15.38±2.87c	ND	14.01±2.79c	ND	147.59±19.63a
(*E*)-2-Decenal	1264	1265	ND	ND	ND	ND	ND	49.66±17.2a
(*E*)-2-Nonenal	1162	1162	ND	ND	ND	ND	ND	60.73±11.29a
(*E*)-Hept-2-enal	957	956	ND	ND	ND	22.08±7.23b	ND	63.86±19.42a
Decanal	1208	1206	3.07±0.64b	7.58±1.29b	1.70±0.28b	5.72±1.44b	1.89±0.23b	81.54±7.73a
2-Undecenal	1366	1367	ND	ND	ND	ND	ND	121.71±23.42a
2-Butyl-2-octenal	1376	1378	ND	ND	ND	ND	ND	117.75±27.44a
Heptanal	900	901	ND	21.95±3.23b	ND	24.06±4.12b	ND	172.87±13.94a

Octanal	1005	1006	ND	16.57±2.71b	ND	ND	ND	204.38±16.70a
(*E*)-2-Octenal	1060	1059	ND	ND	ND	36.53±11.11b	ND	213.97±59.38a
Benzaldehyde	961	960	124.8±37.85b	49.78±8.88c	ND	297.57±18.60a	12.05±2.21c	332.41±26.11a
Nonanal	1106	1107	24.10±5.01c	95.66±16.42b	29.69±7.98c	133.16±29.59b	11.50±1.88c	1391.12±58.71a
Hexanal	789	800	ND	147.15±21.94c	33.03±10.17c	573.82±173.44b	2.90±0.68c	1441.81±197.63a
**Total**			**200.50±36.29cd**	**373.43±58.64c**	**64.42±16.64cd**	**1111.06±208.45b**	**28.34±4.67d**	**4423.57±317.53a**
Esters (4)								
Phenethyl acetate	1260	1258	ND	ND	ND	ND	10.96±2.65a	ND
Methylpropyl propanoate	881	869	ND	ND	14.21±1.68a	ND	ND	ND
Butyl butanoate	998	999	ND	ND	6.21±2.15b	13.22±4.93a	ND	ND
Pentylhexanoate	1291	1291	ND	ND	ND	ND	ND	119.38±10.14a
**Total**			**ND**	**ND**	**20.42±3.81b**	**13.22±4.93b**	**10.96±2.65b**	**119.38±10.14a**
Acids (6)								
Heptanoic acid	1086	1078	ND	ND	ND	ND	ND	18.26±0.63a
2-Methylbutanoic acid	852	861	ND	ND	ND	11.75±1.26b	ND	20.90±2.30a
Isovaleric acid	842	842	ND	ND	ND	31.31±3.55b	ND	52.81±8.41a
Nonanoic acid	1276	1273	ND	ND	ND	ND	ND	72.24±21.01a

Pentanoic acid	894	903	ND	ND	ND	ND	ND	129.90±14.71a
Octanoic acid	1181	1180	ND	ND	ND	ND	ND	131.59±29.77a
**Total**			**ND**	**ND**	**ND**	**43.06±3.19b**	**ND**	**425.70±30.29a**
Phenols (1)								
Phenol	989	980	ND	ND	ND	ND	102.13±25.18a	ND
**Total**			**ND**	**ND**	**ND**	**ND**	**102.13±25.18a**	**ND**

RI[1], retention index calculated by the retention times of n-alkane standard series. RI[2], retention index obtained from the NIST 14. ND, not detected. Different lowercase letters in the same row indicated significant difference ($p < 0.05$).

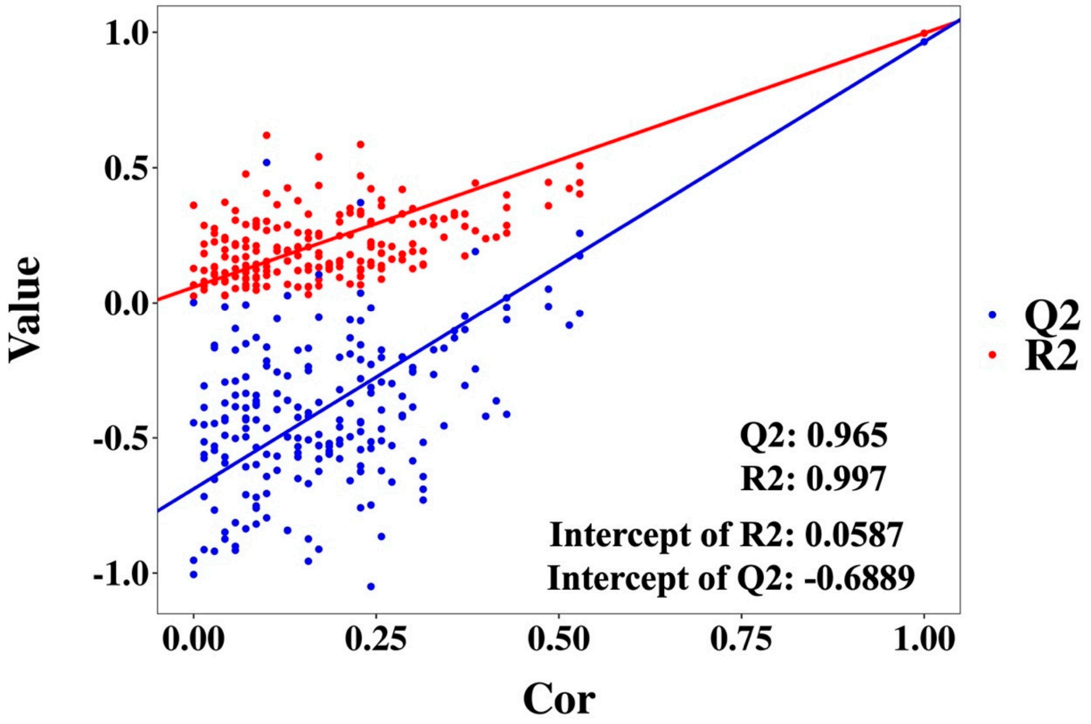

Figure S1. R^2 and Q^2 of PLS-DA analysis of volatile compounds.

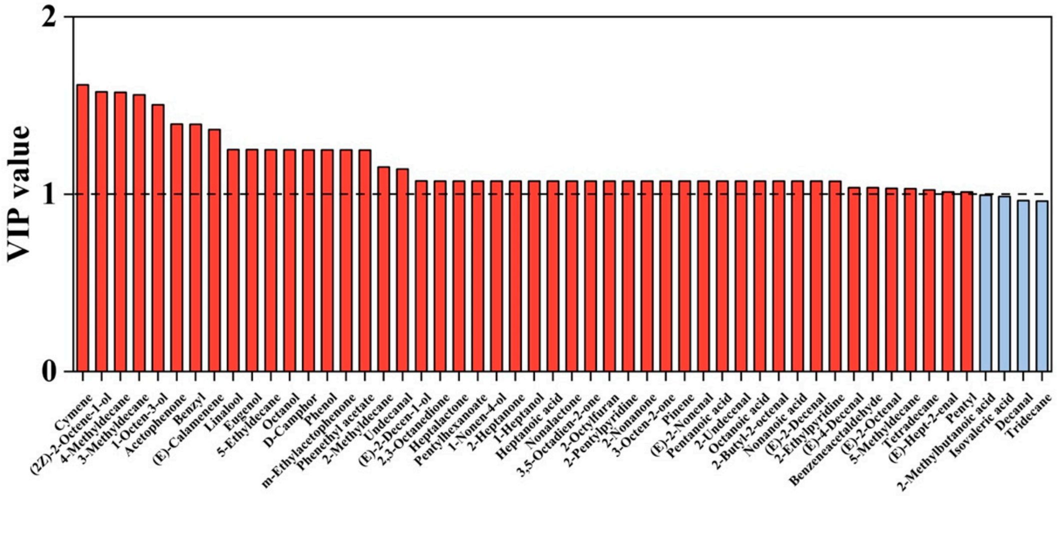

Figure S2. VIP score plots of volatile compounds (top 48) based on PLS-DA analysis.

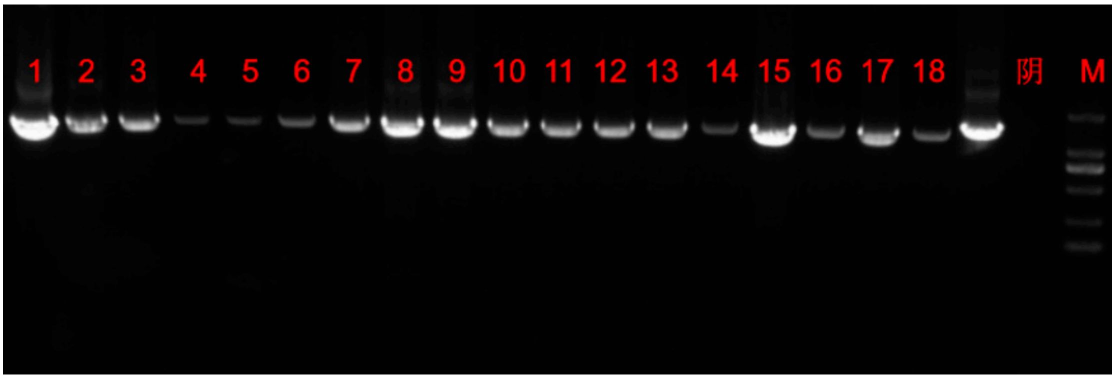

Figure S3. Agarose gel electrophoresis image.

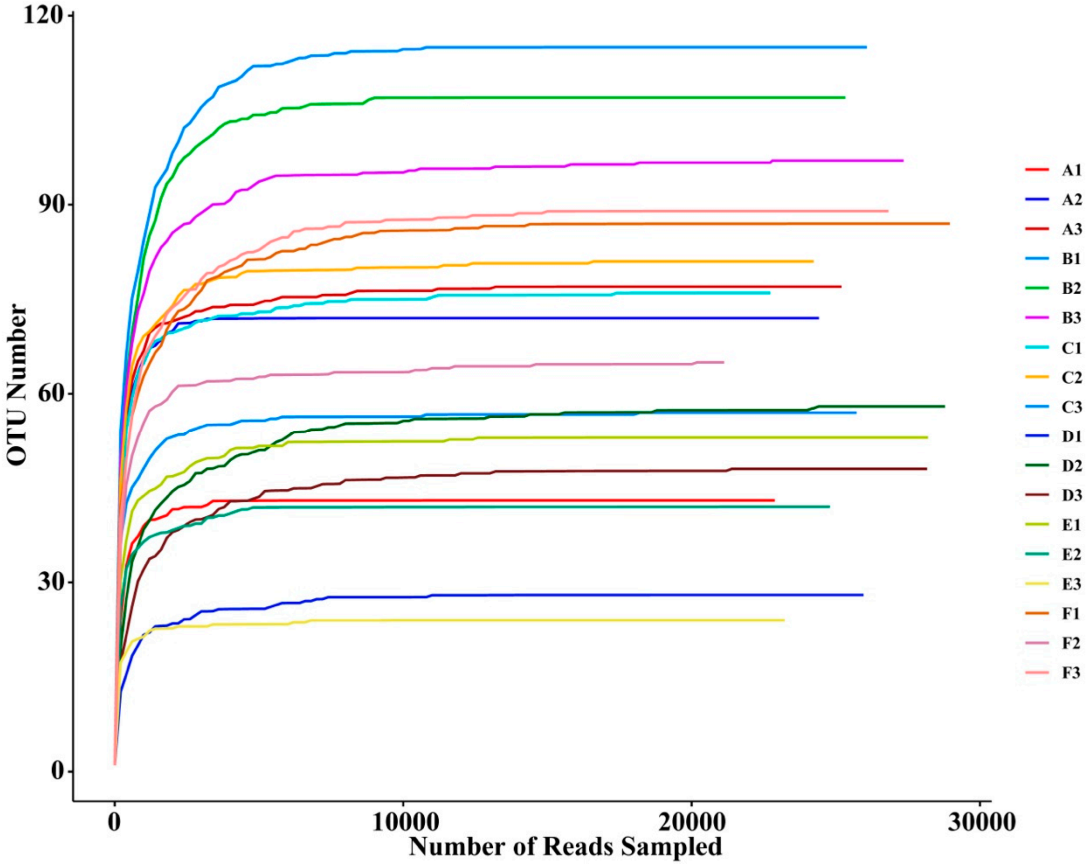

Figure S4. The change in ASV numbers with the sequencing depth.

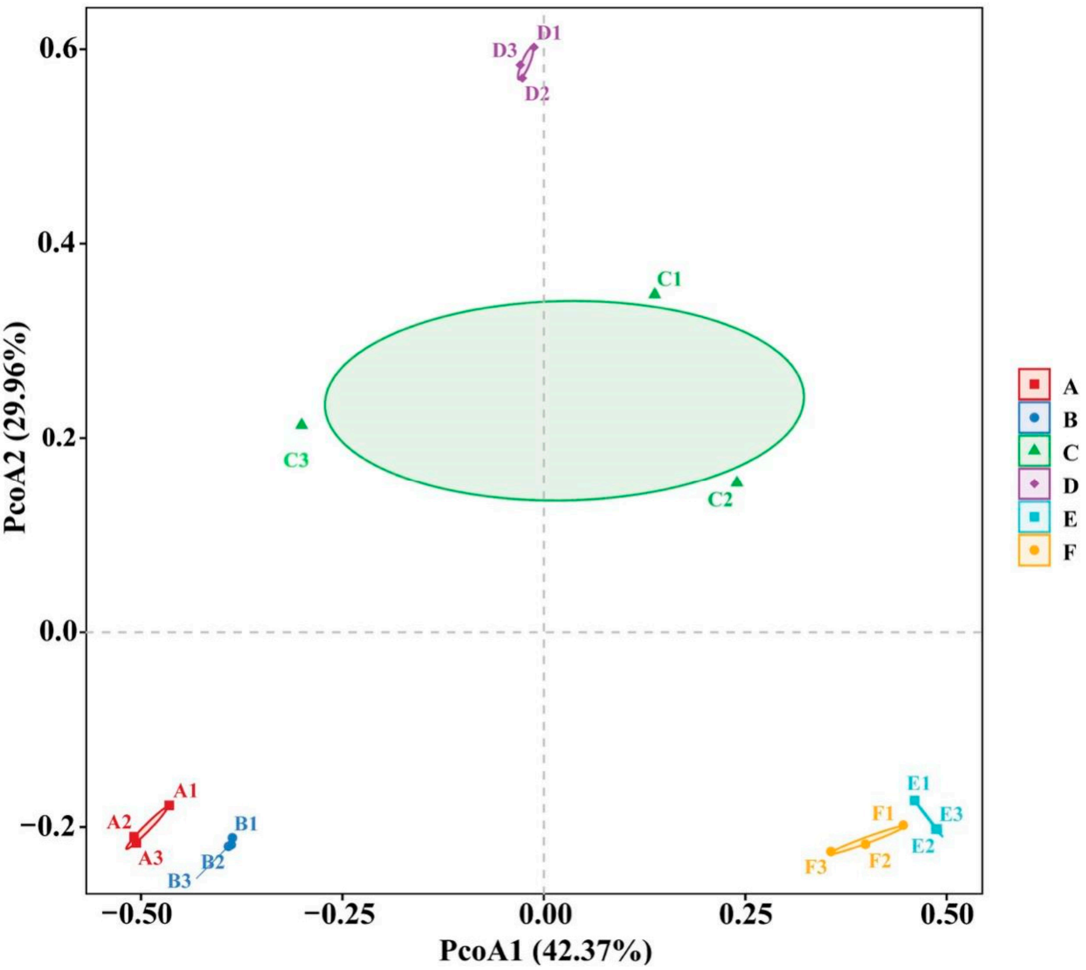

Figure S5. PCoA analysis based on microbial community distances.

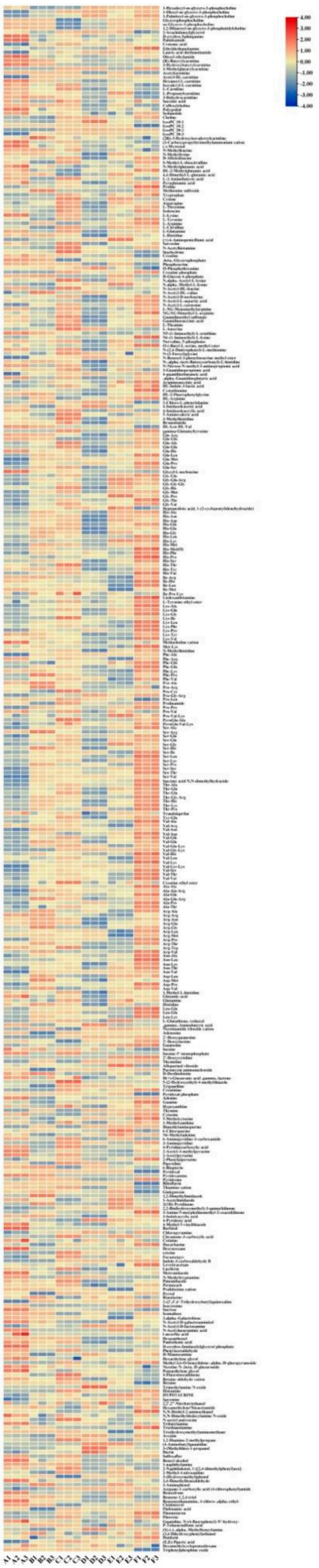

Figure S6. Heatmap visualization of 380 metabolites in grass carp.

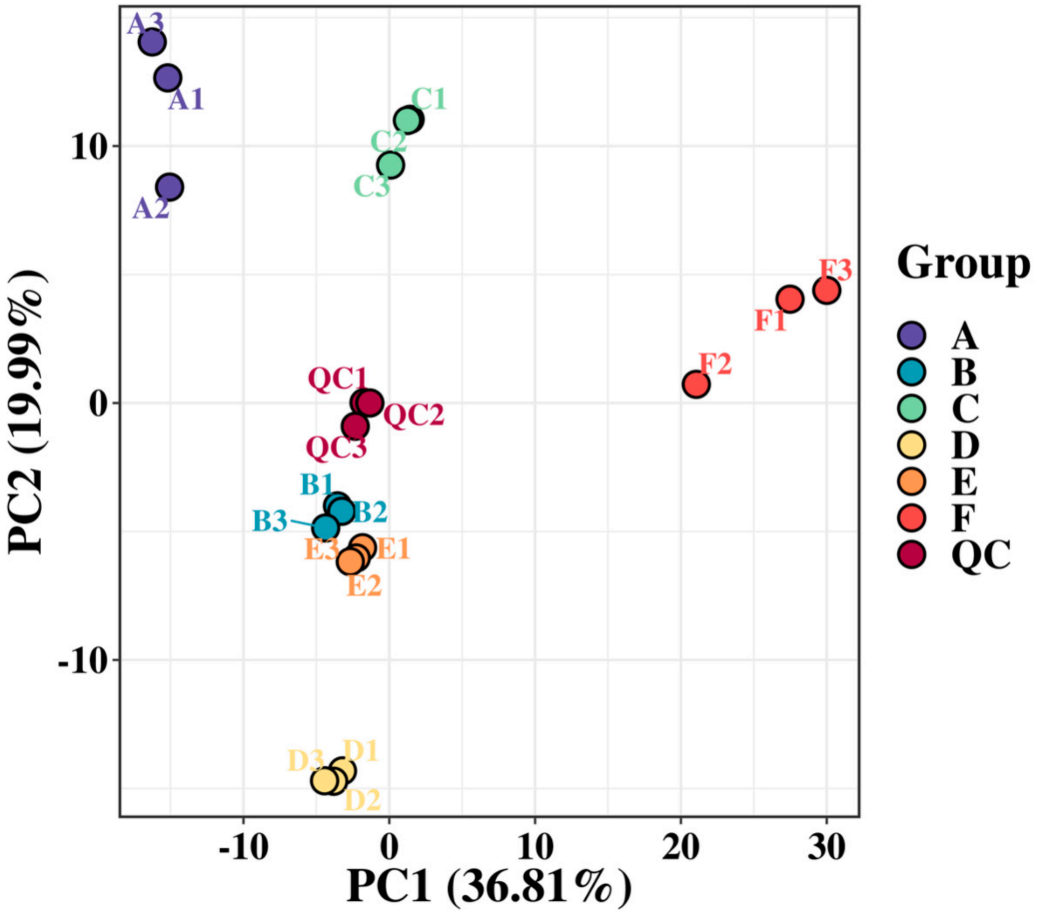

Figure S7. PCA scores plot of metabolites between grass carp samples and QC samples.

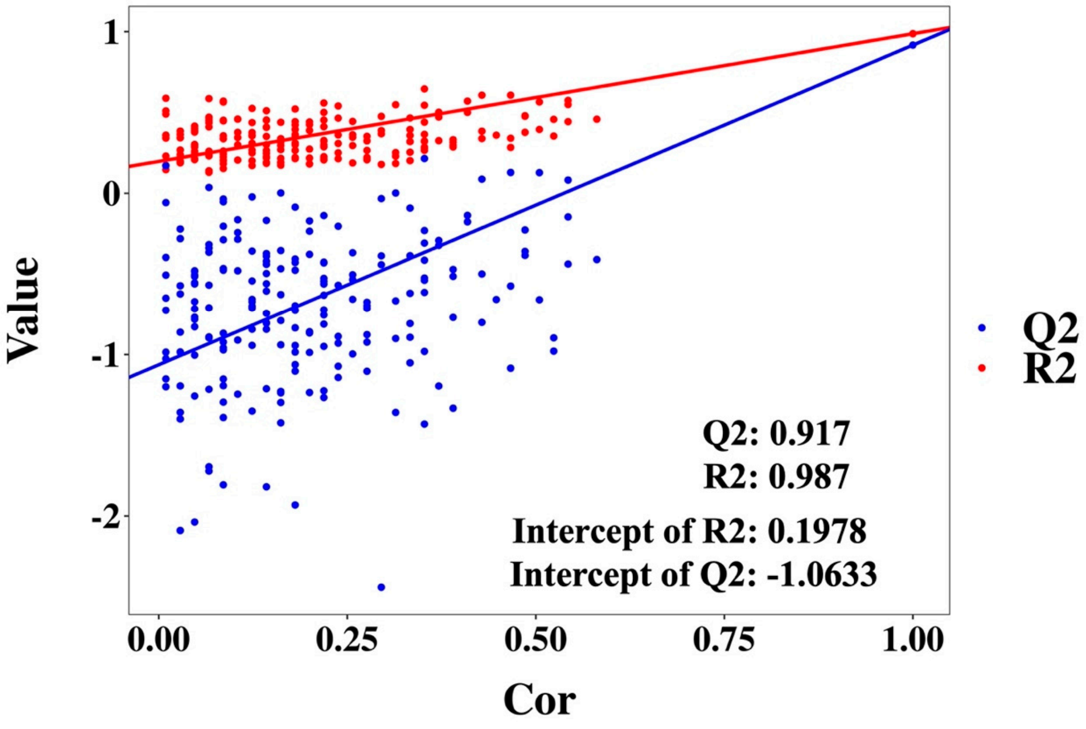

Figure S8. R^2 and Q^2 of PLS-DA analysis of non-volatile metabolites.

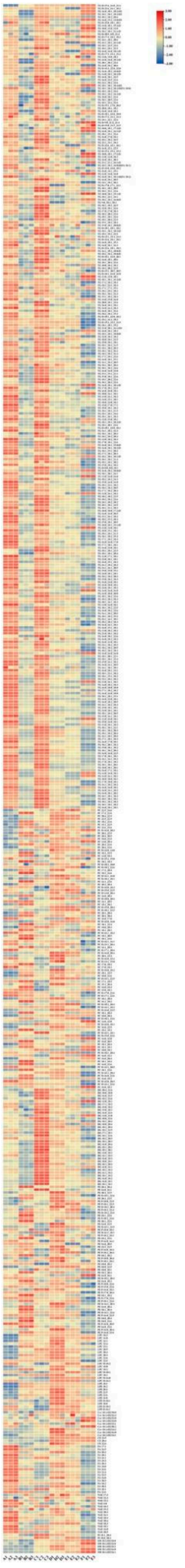

Figure S9. Heatmap visualization of 543 lipid molecules in grass carp.

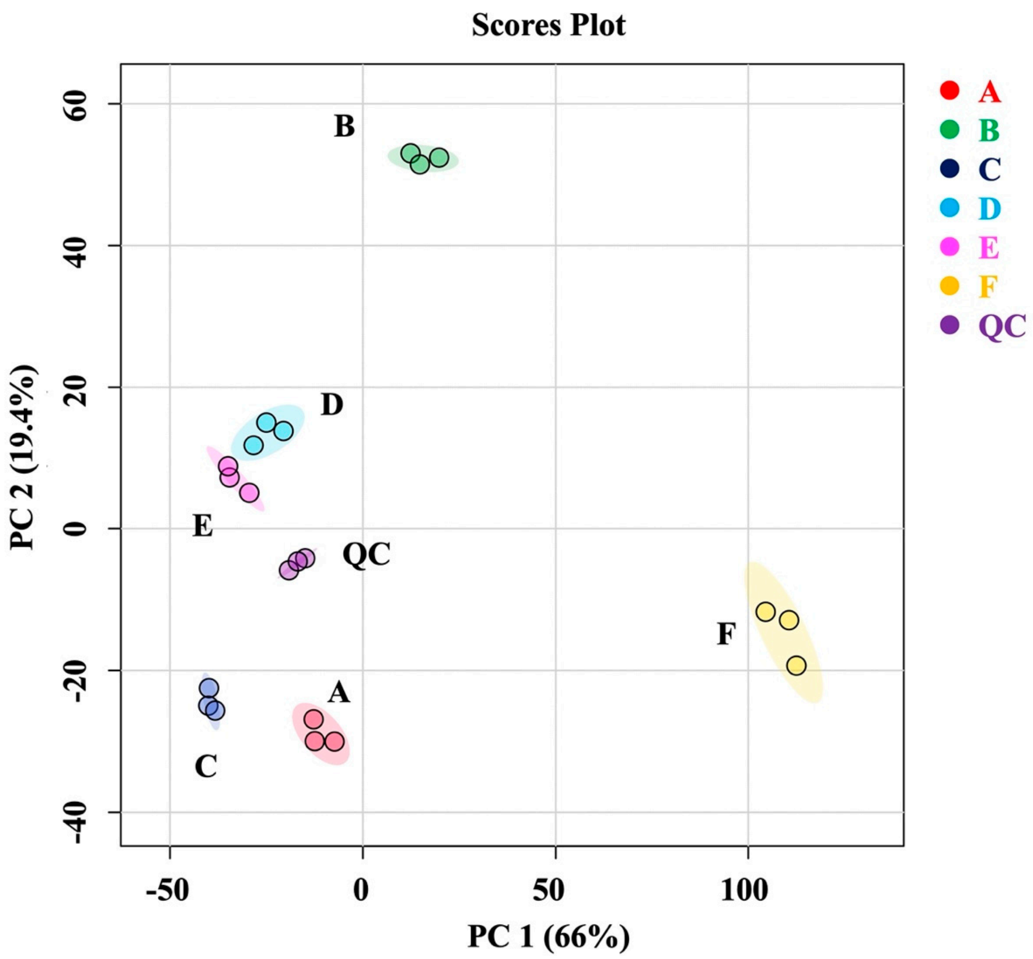

Figure S10. PCA scores plot of lipid molecules between grass carp samples and QC samples.

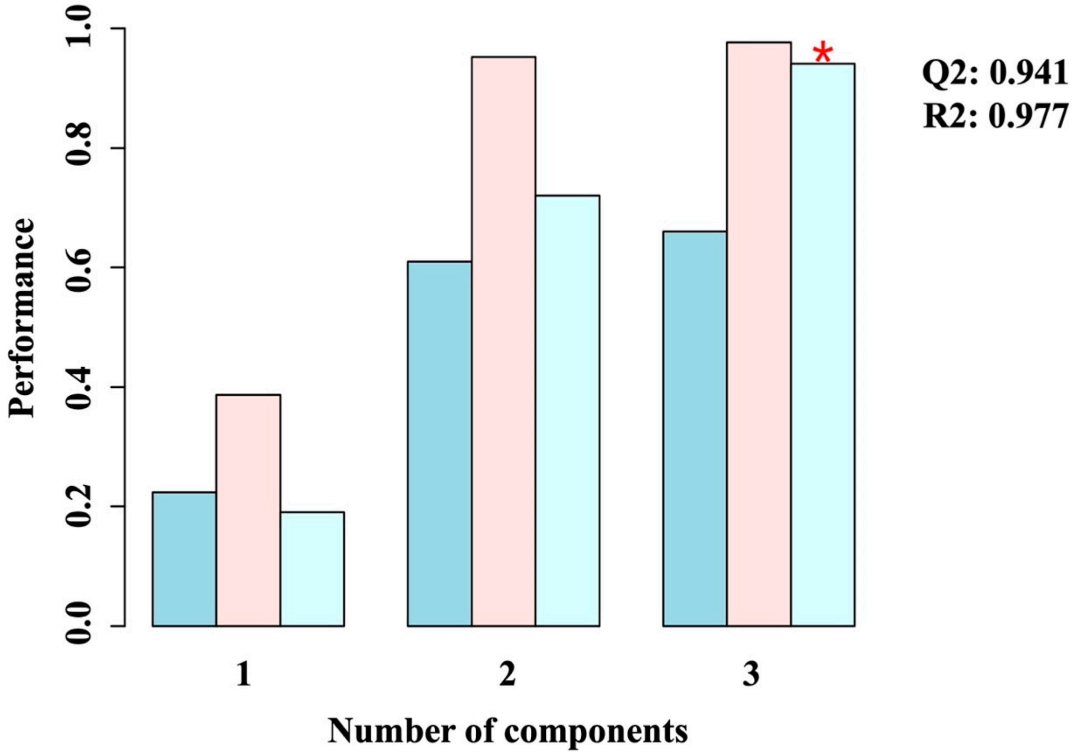

Figure S11. R^2 and Q^2 of PLS-DA analysis of lipid profile.
